# *Cis*-regulatory *CYP6P9b* P450 variants associated with loss of insecticide-treated bed net efficacy against *Anopheles funestus*

**DOI:** 10.1038/s41467-019-12686-5

**Published:** 2019-10-11

**Authors:** Leon M. J. Mugenzi, Benjamin D. Menze, Magellan Tchouakui, Murielle J. Wondji, Helen Irving, Micareme Tchoupo, Jack Hearn, Gareth D. Weedall, Jacob M. Riveron, Charles S. Wondji

**Affiliations:** 1 Vector Biology Department, Liverpool School of Tropical Medicine, Pembroke Place, Liverpool, L3 5QA UK; 2 Centre for Research in Infectious Diseases (CRID), P.O. Box, 13501 Yaoundé, Cameroon; 3 Department of Biochemistry and Molecular Biology, Faculty of Science University of Buea, P.O. Box, 63 Buea, Cameroon; 4 School of Natural Sciences and Psychology, Liverpool John Moores University, Byrom Street, Liverpool, L3 3AF UK

**Keywords:** Genetic markers, Parasitic infection, Parasitology, Malaria

## Abstract

Elucidating the genetic basis of metabolic resistance to insecticides in malaria vectors is crucial to prolonging the effectiveness of insecticide-based control tools including long lasting insecticidal nets (LLINs). Here, we show that *cis*-regulatory variants of the cytochrome P450 gene, *CYP6P9b*, are associated with pyrethroid resistance in the African malaria vector *Anopheles funestus*. A DNA-based assay is designed to track this resistance that occurs near fixation in southern Africa but not in West/Central Africa. Applying this assay we demonstrate, using semi-field experimental huts, that CYP6P9b-mediated resistance associates with reduced effectiveness of LLINs. Furthermore, we establish that *CYP6P9b* combines with another P450, *CYP6P9a*, to additively exacerbate the reduced efficacy of insecticide-treated nets. Double homozygote resistant mosquitoes (RR/RR) significantly survive exposure to insecticide-treated nets and successfully blood feed more than other genotypes. This study provides tools to track and assess the impact of multi-gene driven metabolic resistance to pyrethroids, helping improve resistance management.

## Abstract (web summary)

Bed nets treated with insecticides have been instrumental in reducing malaria mortality, but insecticide resistance is on the rise. Here, Mugenzi et al. identify genetic variants in the P450 gene *CYP6P9b* of *Anopheles funestus* that associate with insecticide resistance and develop a PCR-based diagnostic assay to help identify pyrethroid-resistant strains.

## Introduction

Malaria remains a major public health burden in Africa. Control strategies rely predominantly on insecticide-based interventions such as indoor residual spraying and long-lasting insecticide nets (LLINs). These tools have been estimated to be responsible for more than 68% of the decrease in malaria mortality in the past 15 years having helped prevent more than 663 million clinical cases of malaria^1^. Increasing insecticide resistance in malaria vector species presents a major challenge to the continued success of public health interventions. The elucidation of the molecular basis of insecticide resistance in these vectors and its evolution across Africa is a crucial step to design resistance management strategies to prevent potentially devastating public health consequences. Detailed information on resistance mechanisms is a prerequisite to detect resistance markers facilitating the design of field-applicable diagnostic tools. Unlike current WHO bioassays that only detect resistance once it is well established in the population^2^, these molecular diagnostic tools can detect and track resistance at an early stage, which is an essential requirement of resistance management efforts.

Resistance to insecticides is primarily caused by two major mechanisms: target-site resistance (e.g., knockdown resistance, *kdr*) and metabolic resistance through elevated expression of detoxification genes, especially the cytochrome P450s^3,4^. Target-site resistance through knockdown resistance (*kdr*) mutations in the voltage-gated sodium channel gene is well characterised with DNA-based diagnostic tools designed since the late 1990s^5,6^.

In contrast, metabolic resistance, considered to be more likely to cause control failure^7^, remains—despite recent progress^8–11^—poorly characterised due to the complexity of this resistance mechanism^12^. This is the reason why although several cytochrome P450 genes associated with pyrethroid resistance have been reported, only a single DNA-based marker has been detected to date^13^ limiting the design of field applicable diagnostic tools to detect and track this resistance. Indeed, progress was recently made with the detection of the first DNA-based P450 resistance marker in mosquitoes in the *CYP6P9a* gene on the *rp1* (resistance to pyrethroid 1) quantitative trait locus (QTL)^14,15^ allowing assessment of the impact of metabolic resistance on the effectiveness of insecticide-treated nets^13^.

However, the *CYP6P9a* marker does not explain all of the genetic variance in pyrethroid resistance and is currently only present in southern Africa^13,16,17^. Thus, there is a need to characterise the molecular basis of pyrethroid resistance Africa-wide and detect related genetic variants so that DNA-based diagnostic assays can be designed to enhance our ability to detect resistance and rigorously characterise the impact on insecticide-based interventions.

To tackle the molecular complexity of metabolic resistance to pyrethroids and detect resistance markers and design reliable diagnostic assays, we carry out large scale transcriptional and genomic profiling of *Anopheles funestus* mosquitoes across Africa. Our results reveal the complex and diverse molecular changes underlying insecticide resistance across the species’ range characterised by differential gene expression patterns and genomic signatures, notably transcription factor binding sites (*cis*-acting regulatory elements) regulating expression of detoxification genes. This thorough elucidation of metabolic resistance leads to the detection of a P450 DNA-based marker in the *CYP6P9b* gene from which a simple field-applicable polymerase chain reaction (PCR) diagnostic assay is designed. Field assessment of the impact of pyrethroid resistance using this marker in semi-field conditions in experimental huts revealed that this metabolic resistance marker in combination with the other P450 marker (*CYP6P9a*) is associated with exacerbated loss of efficacy of insecticide-treated nets.

## Results

### Transcription analysis of resistant *An. funestus* Africa-wide

Previous efforts to detect genes associated with pyrethroid resistance Africa-wide have compared mosquitoes alive after permethrin exposure to the lab-susceptible FANG strain only^13^. A limitation of this approach is that the over-expressed genes might not be necessarily linked to resistance but rather other differences in genetic background. Therefore, to detect potential genes associated with pyrethroid resistance across Africa [Southern (Malawi), East (Uganda), Central (Cameroon) and West (Ghana)], we used here two additional approaches by first comparing permethrin-resistant mosquitoes from one country to permethrin-resistant mosquitoes from another country to detect the genes specific for each region of Africa. Second, we performed in Malawi a triangular comparison of the expression patterns of mosquitoes alive after exposure (R) to those not exposed (Control; C) as well as the lab-susceptible strain FANG (S).

Comparisons of the transcription profiles between the four African regions were performed to detect or confirm genes differentially expressed according to their resistance background. When the southern Africa population (Malawi) is compared to all other regions directly, the striking difference is the high up-regulation of two P450 genes *CYP6P9a* and *CYP6P9b* in Malawi with fold change (FC) of 28.3, 22.7 and 9.5 greater than in Cameroon, Uganda and Ghana for *CYP6P9a* (Supplementary Data 1). Both genes result from a recent duplication event^14^. A similar but lower difference is observed for *CYP6P9b* with FC values of 5.5, 3.4 and 3.5, respectively. In contrast to *CYP6P9b, CYP6P9a* is also significantly more over-expressed in Ghana compared to Cameroon and Uganda. The southern Africa mosquito population has more genes significantly up-regulated than other regions including cytochrome P450s, *CYP4H25, CYP4H28, CYP9J11* (except Ghana) and *CYP6P2* (except Cameroon) although the FC is much lower than for the duplicated genes. Two cuticular protein genes, potentially involved in cuticular resistance (AFUN005672 and AFUN009940) were also significantly over-expressed in Malawi compared to the three other regions (Supplementary Data 1).

The biggest difference between the West African sample of Ghana and others was the duplicated *CYP6P4a* and *CYP6P4b* that are significantly more up-regulated in Ghana than other regions with FC values of 25.8, 7.6 and 21.2 for *CYP6P4a*, respectively, against Cameroon, Malawi and Uganda or FC values of 22.7, 6.5 and 9 for *CYP6P4b*. These genes are also significantly more expressed in mosquitoes from southern Africa than in Central and East Africa.

The Central African population of Cameroon exhibited a greater difference than other regions for the cytochrome P450 *CYP325A* gene with FC values of 5.3, 12.1 and 4.5 against Malawi, Uganda and Ghana, respectively, although the overall abundance of reads for this gene is lower (<500). The carboxylesterase gene AFUN002514 was also significantly overexpressed in Cameroon compared to the south (FC 3.8) and the east (FC 4.7) but not against Ghana where it is also over-expressed compared to Malawi (FC 2.5) and Uganda (FC 3.3). This suggests that the over-expression of the carboxylesterase AFUN002514 is specific to West-Central Africa.

The East African population from Uganda exhibited the greatest difference in expression to all other regions for the cytochrome P450 *CYP9K1* with FC values of 4.6, 2.6 and 1.8 against Cameroon, Malawi and Ghana suggesting that this gene is potentially specific to pyrethroid resistance in the East Africa region. The P450 *CYP307A1* is also up-regulated in Uganda relative to other regions (Supplementary Data 1). Further differences between regions are highlighted in Supplementary Note 1.

**Fig. 1:**
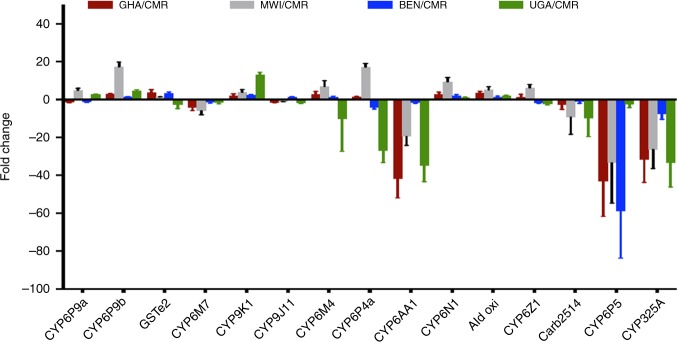
Comparative transcription profiles of *An. funestus* between countries. qRT-PCR comparison of the expression profile of major detoxification genes associated with pyrethroid resistance between populations of *An. funestus* from different African regions supporting a shift in the role of these genes between regions. The data shown are mean + SEM (*n* = 3). Source data are provided as a Source Data file

In addition, a triangular analysis of transcriptional profiles between resistant (R), unexposed control (C) and susceptible (S) mosquito samples in Malawi confirmed the major role potentially played by *CYP6P9a* and *CYP6P9b* in these countries with a slightly higher level in R–S compared to C–S (FC 60.1 vs. 57.1 for *CYP6P9a*; FC 23.7 vs. 18.5 for *CYP6P9b*) (Supplementary Table 1; Supplementary Note 2). Moreover, the quantitative reverse transcription (qRT) PCR expression patterns broadly supported the differences observed with RNAseq in the main genes driving pyrethroid resistance in different African regions (Fig. 1; Supplementary Note 3).

### Genetic polymorphisms of CYP6P9b-mediated resistance

To detect potential genetic variants associated with pyrethroid resistance, we focused our attention on the *CYP6P9b* P450 because this gene was highly over-expressed in resistant mosquitoes especially in southern Africa and after the recent characterisation of *CYP6P9a*, the other over-expressed gene in southern Africa^13^.

To have a full view of the potential regulatory elements driving the over-expression of *CYP6P9b*, we amplified and sequenced the full 1 kb intergenic region (Supplementary Fig. 1; Supplementary Note 4) between *CYP6P9b* and *CYP6P5* in individual resistant (FUMOZ_R) and susceptible (FANG) mosquitoes. An indel of 3 bp (AAC) was consistently observed with deletion in the resistant FUMOZ and presence in susceptible FANG. The core promoter elements detected using GPminer include TATA boxes (8 in FUMOZ and 7 in FANG and 1 GC box in each strain), 1 GC box and 1 CCAAT box. The *CYP6P9b* promoter also exhibits two sites for the putative arthropod initiator (Inr) contrary to one for the 800 bp promoter fragment of *CYP6P9a* previously studied^13^. Furthermore, several transcription factor binding sites were detected including six sites for the CncC nrf2/MAF (in both strains) and several sites for GATA, MYB or AHR transcription factors.

The polymorphism pattern of the promoter region of *CYP6P9b* was also assessed Africa-wide by sequencing the 1 kb intergenic region in mosquitoes from West, Central, East and Southern Africa. All populations from southern Africa (Mozambique, Malawi and Zambia) exhibited a low genetic diversity with Mozambique presenting the least diverse set with a number of haplotype (h) = 2 and a haplotype diversity (hd) = 0.189. However, the population of Benin (West Africa) exhibited no segregating sites with only a single haplotype observed. This absence of polymorphism in Benin (similar for *CYP6P9a*) contrasts highly with neighbouring populations notably that of Nigeria which exhibits the highest diversity (40 segregating sites, h = 13; hd = 0.95) (Supplementary Table 2).

**Fig. 2:**
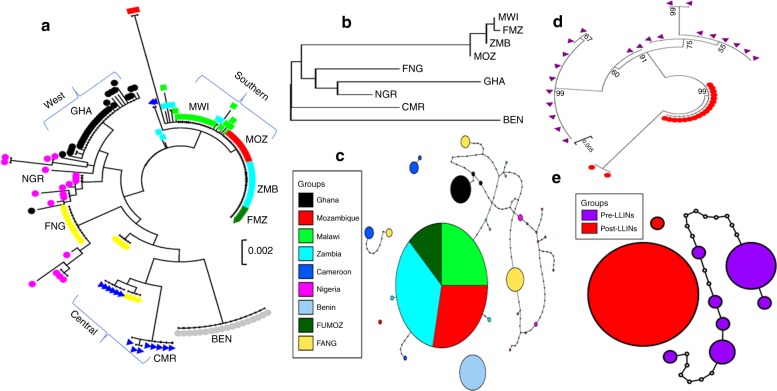
Genetic diversity patterns of the *cis*-regulatory region of *CYP6P9b*. **a** Maximum likelihood phylogenetic tree of *CYP6P9a* promoter haplotypes across Africa. **b** Neighbour-joining phylogenetic tree of CYP6P9b-based genetic distance between nine African populations (N_ST_ estimates). **c** Africa-wide TCS network for the *CYP6P9b* haplotypes showing predominant haplotypes in southern Africa. A fixed haplotype is observed in Benin (sky blue) and a nearly fixed haplotype is seen in Ghana (black). Other locations show a greater diversity. Lines connecting haplotypes and each node represent a single mutation event. **d** A maximum likelihood phylogenetic tree of *CYP6P9b* showing a cluster of highly diverse haplotypes before scale up of bed nets (pre-intervention) but a nearly fixed haplotype post-intervention. **e** TCS haplotype network in Mozambique pre- and post-intervention revealing a major resistant haplotype post-intervention but a more diverse set of haplotypes before pre-bed nets

Overall, southern Africa populations consistently exhibited a different polymorphism pattern to other regions notably with the presence of an AAC deletion 50 bp upstream of nrf2/MAF binding sites and 72 bp from a CCAAT box. The AAC deletion located 703 bp from start codon is tightly associated with other polymorphisms in a haplotype (STH13) (Supplementary Fig. 2). This haplotype predominates in southern Africa (68/82) reflecting the marked selective sweep observed around this gene in this region^18^. A maximum likelihood phylogenetic tree of all haplotypes (Fig. 2a) revealed three broad clusters corresponding to southern Africa, West and Central Africa; the unique Benin haplotype is different from other West Africa haplotypes and is closer to the Central Africa Cluster (Fig. 2a).

The difference in genetic diversity between southern Africa and other regions was further supported by the higher genetic differentiation between these populations as shown by the neighbour-joining tree of the genetic distance (*N*_*ST*_ estimates) (Fig. 2b). A haplotype network built with the Templeton, Crandall and Sing (TCS) programme, further highlights the reduced diversity in southern Africa with the predominant STH13 haplotype in all populations from this region (Fig. 2c); greater diversity is seen in other samples with a high number of mutational steps between haplotypes observed particularly in Nigeria. Nevertheless, other countries such as Benin and Ghana also have a predominant haplotype suggesting possible selection also acting on this gene or in nearby genes in these locations.

To assess whether the differences observed in the 5’UTR region of *CYP6P9b* between southern Africa and other regions could have resulted from selection due to insecticide pressure, we assessed the polymorphism of the 1 kb fragment of *CYP6P9b* in samples collected before the scale-up of LLINs in Mozambique (2002) and samples collected after scale-up (2016). This 1 kb fragment was polymorphic before scale-up with many segregating sites (18) and haplotypes (h = 6; hd = 0.797) as well as high nucleotide diversity (*π* = 0.011) (Supplementary Table 2; Fig. 2d). In contrast, the post-intervention samples exhibited very low diversity with low haplotypes number (h = 2; hd = 0.189) and reduced nucleotide diversity (*π* = 0.0053). Construction of the maximum likelihood tree further highlights the contrast between both sets of samples as the pre-intervention mosquitoes cluster together and become more diverse (Fig. 2d). In contrast, the post-intervention samples cluster noticeably away from the pre-intervention with a drastically reduced haplotype number. The TCS haplotype network further supported the drastic difference between pre- and post-intervention samples with more mutational steps observed between pre-intervention haplotypes whereas a highly predominant haplotype is observed in post-intervention samples (18/20). This is not observed in the pre-intervention samples, which is probably an indication of its low frequency before bed nets (Fig. 2e). Furthermore, the AAC motif now undetectable in the southern populations and located 50 bp upstream of a nrf2/MAF binding site was present in all pre-intervention samples suggesting that this deletion plays a role in the regulation of *CYP6P9b*. These major differences between pre- and post-intervention samples show that scale up of insecticide-treated nets is likely a major factor that has driven this evolution in *An. funestus* populations in southern Africa.

### *CYP6P9b* promoter analysis detects resistance variants

**Fig. 3:**
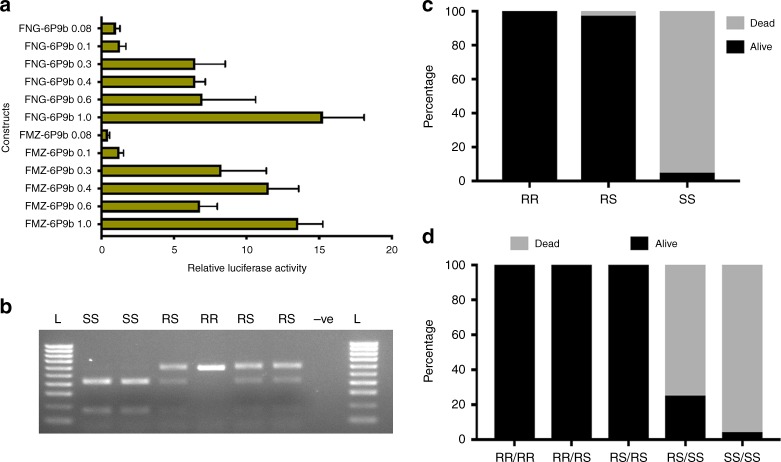
DNA-based diagnostic assay for CYP6P9b-mediated metabolic resistance to pyrethroids. **a** Comparative luciferase assay between promoter fragment from the highly resistant FUMOZ and highly susceptible (FANG) lab strains with progressive serial deletions of *CYP6P9b* 5′ flanking region to detect the causative variants. Bars represent the mean ± S.D. of four independent transfections of three replicates (*n* = 6). **b** Agarose gel of PCR-RFLP with *Nmu*Cl of *CYP6P9b* showing the RR, RS, and SS genotypes. **c** Distribution of the *CYP6P9b* genotypes between susceptible and resistant mosquitoes showing a very strong correlation between *CYP6P9b* and resistance phenotype. **d** Distribution of the combined genotypes of both *CYP6P9a* and *CYP6P9b* showing that both genotypes combined to increase the pyrethroid resistance. Source data are provided as a Source Data file

The 1 kb fragment upstream of the translation start site of *CYP6P9b* was assessed for its promoter activity using a luciferase assay in the *An. gambiae* 4a-2 cell line. To narrow down the regions containing the potential regulatory motifs, six different sized fragments of the 1 kb, 600 base pairs (bp), 400 bp, 300 bp, 100 bp and 80 bp immediately upstream of the translation start codon from both the resistant FUMOZ and the susceptible FANG strain were cloned upstream of a reporter gene in a pGL3 vector. These constructs were used in luciferase reporter gene assays but did not show a significant difference in the relative luciferase activity of the 1 kb insert between the two strains. Similarly, the shorter versions of the putative promoter did not present a significantly different activity between these two strains. Nevertheless, the progressive truncation of the 1 kb insert helped identify the major regulatory elements in either strain. Shortening the promoter fragment to 600, 400 and 300 bp reduced their activity in FUMOZ by 49.9 (*P* < 0.0001; unpaired *t* test), 21.1 (*P* = 0.0074; unpaired *t* test) and 39.0% (*P* = 0.0043; unpaired *t* test) respectively, and similarly in FANG with reductions of 54.4 (*P* = 0.0014; unpaired *t* test), 57.6 (*P* < 0.0001; unpaired *t* test) and 57.7 (*P* < 0.0001; unpaired *t* test), respectively (Fig. 3a). However, for both strains, a much higher reduction in luciferase activity is seen when the promoter fragment is further truncated to just 100 (>91%) and 80 bp (>93%) (*P* < 0.0001; unpaired *t* test) with deletion of all regulatory elements including CncC nrf2/MAF and CCAAT boxes (Fig. 3a).

### A DNA-based diagnostic tool to detect *CYP6P9b* resistance

To design a DNA-based diagnostic assay to detect *CYP6P9b*-mediated resistance, we screened the 1 kb for suitable restriction sites in complete linkage with the AAC deletion in the resistant mosquitoes across southern Africa including in the FUMOZ strain (Supplementary Fig. 3a). This allowed the design of a simple PCR restriction fragment length polymorphism (RFLP). We found a restriction site for the *Nmu*Cl (*Tsp*45I) (cut site 5′-GTSAC-3′) spanning a C/T mutation located 11 bp downstream of the AAC deletion and designed a PCR-RFLP that, in mosquitoes carrying the *CYP6P9b* variants associated with susceptibility to insecticides, cuts the 550 bp amplicon in two fragments of 400 and 150 bp, whereas the amplicon carrying the resistance-associated allele remains uncut (Fig. 3b; Supplementary Fig. 3b). To assess the efficacy of this diagnostic assay, we genotyped the FUMOZ-R and FANG strains for the CYP6P9b-R marker and establish that all 50 tested FUMOZ-R mosquitoes were homozygote resistant RR whereas all the FANG were homozygote susceptible SS.

To further validate the ability of CYP6P9b_R marker to predict pyrethroid resistance phenotype in *An. funestus*, mosquitoes were crossed between FUMOZ and FANG at the F_8_ generation. Genotyping of the same mosquitoes previously used to validate the *CYP6P9a*^13^ revealed that those surviving exposure to permethrin for 180 min are predominantly homozygote resistant (10/48) and heterozygotes (36/48) with two bearing the homozygote susceptibility genotype. From the 47 dead mosquitoes after 30 min exposure to permethrin (highly susceptible), 46/47 were homozygote susceptible and one heterozygote (Supplementary Fig. 3c). A significant association was observed between permethrin resistance and the *CYP6P9b* genotype with a highly significant odds ratio when comparing RR vs. SS (OR = infinity; *P* = 1.1 × 10^−50^, Fisher’s exact test) and RS vs. SS (OR = 715; *P* = 2.4 × 10^−45^, Fisher’s exact test) (Fig. 3c).

The contribution of *CYP6P9b* to pyrethroid resistance was also compared to that of *CYP6P9a* revealing that both genes combine additively to confer higher level of resistance. Indeed, all mosquitoes with the double homozygote resistant genotypes (RR/RR) were alive after 180 min exposure. However, only half of the homozygous resistance-genotype carriers for *CYP6P9b* also had the homozygous resistant genotype for *CYP6P9a* with the other half being only heterozygous for *CYP6P9a* (Supplementary Fig. 3d). 66.7% of mosquitoes that survived were double heterozygous (RS/RS), whereas only 10.4% were RR/RR carriers and 16.7% RR/RS carriers (Fig. 3d: Supplementary Fig. 3d) suggesting possible fitness cost associated with possessing double resistance alleles for both genes. None of the dead mosquitoes had RR/RR, RR/RS or RS/RS genotypes; this shows a strong correlation with permethrin resistance with high OR (infinity; *P* < 0.0001, Fisher’s exact test).

### Distribution of the resistant *CYP6P9b* allele across Africa

**Fig. 4:**
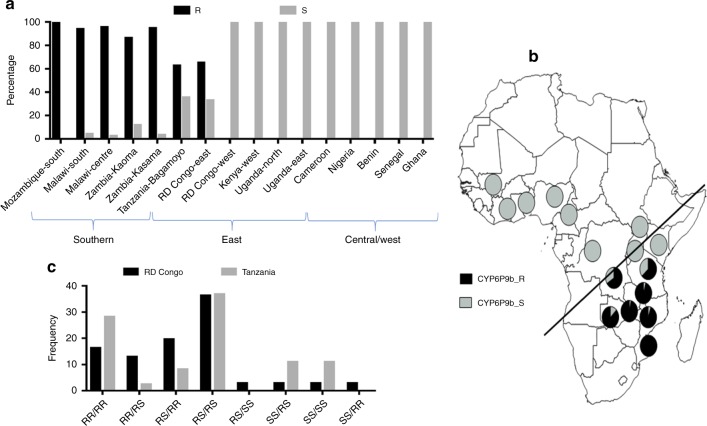
Geographical distribution of *CYP6P9b* Africa-wide. **a** Frequency of the CYP6P9B_R allele across Africa showing that it is highly predominant in southern Africa, moderately present in East Africa but completely absent elsewhere on the continent. **b** Map of Africa showing the distribution of the CYP6P9b-resistant alleles with a near fixation in southern Africa. The map was generated using a blank map freely available from https://www.rkkerkenschijndel.nl/map-of-africa-drawing.html and pie-charts representing frequency of both alleles in each location were added on the map. **c** Comparative distribution of the combined genotypes of *CYP6P9a* and *CYP6P9b* in Tanzania and RD Congo supporting an independent segregation of genotypes of both genes in the field. Source data are provided as a Source Data file

Genotyping of the *CYP6P9b* was successful in most countries, but some samples failed to amplify in Kenya, Uganda, Cameroon and DR Congo (western region only). It is possible that a structural variant is present in these countries preventing the amplification of the 550 bp fragment. The *CYP6P9b* resistant allele (CYP6P9b_R) is detected throughout southern Africa at a very high frequency (>90%) with fixation observed in Mozambique (Fig. 4a), consistent with previous results from *CYP6P9a*^19^. An intermediate frequency is observed in Tanzania (63.4% for CYP6P9b_R) and in Eastern Democratic Republic of Congo (66.6%). CYP6P9b_R is absent from other parts of Africa including East (Kenya, Uganda), Central (Democratic Republic of Congo (DRC)-West, Cameroon) and West Africa (Fig. 4b). Furthermore, the potential association of CYP6P9b_R with CYP6P9a_R in field populations was assessed in Tanzania and DRC where there is segregation of these genotypes. This implies that there is a greater linkage of *CYP6P9a* and *CYP6P9b* genotypes in Tanzania with 71.8% of identical genotypes; this is much lower in DRC where only 51% identical genotypes are observed between the two genes in assessed mosquitoes (Fig. 4c). However, the RS/RS genotype is predominant (>36%) in both countries with double homozygote resistant RR/RR being higher in Tanzania (28.5%) than DRC (16.7%).

### CYP6P9b_R associated with reduced efficacy of insecticide-treated nets

The impact of the *CYP6P9b* resistance allele on the effectiveness of conventional (PermaNet 2.0) and PBO-based (PermaNet 3.0) nets was assessed using a release-recapture of mosquitoes from the hybrid strain between highly resistant FUMOZ-R and fully susceptible FANG as described for *CYP6P9a*^13^. Mosquitoes from FANG/FUMOZ crosses (F_4_) were moderately resistant to pyrethroids with mortality rates of >70% for both permethrin and deltamethrin after 1 h exposure.

**Fig. 5:**
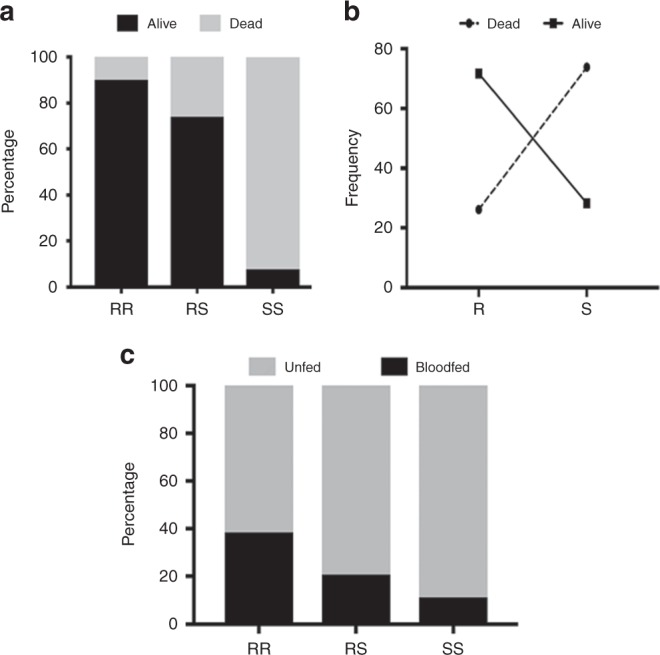
Impact of the *CYP6P9b*-based metabolic resistance on bed nets’ efficacy. **a** Distribution of *CYP6P9b* genotypes between dead and alive mosquitoes after exposure to PermaNet 2.0 net in experimental huts showing that CYP6P9b_R significantly allows mosquitoes to survive exposure to this insecticide-treated net. **b** Association between frequency of CYP6P9b-R and ability to survive exposure to PermaNet 2.0. **c** Distribution of *CYP6P9b* genotypes between blood-fed and unfed mosquitoes after exposure to the PBO-based net PermaNet 3.0 showing that CYP6P9b_R allele increases the ability to take a blood meal. Source data are provided as a Source Data file

To assess the impact of *CYP6P9b* on the efficacy of LLINs, we first analysed this impact on the mortality of mosquitoes after exposure to PermaNet 2.0 because the number of live mosquitoes was too low with PermaNet 3.0. Both nets are impregnated with deltamethrin. The association between *CYP6P9b* genotypes and the ability to survive exposure to PermaNet 2.0 was first assessed using only mosquitoes collected in the room and unfed to avoid possible bias due to feeding or exophily status. This analysis revealed a significant difference in the distribution of genotypes between dead and live mosquitoes (chi square = 1180; *P* < 0.0001, chi-square) (Fig. 5a). Comparing the proportion of different genotypes according to mortality outcome revealed that *CYP6P9b* homozygote resistant (RR) were significantly more able to survive exposure to PermaNet 2.0 than homozygotes-susceptible mosquitoes (OR = 109.3; CI = 40.7–293.2; *P* < 0.0001, Fisher’s exact test) (Table 1). This correlation is even stronger than previously observed for *CYP6P9a* in the same mosquitoes (OR = 34.9; CI = 15.8–77.1; *P* < 0.0001, Fisher’s exact test)^13^.

**Table 1:** Correlation between genotypes of *CYP6P9b* and mortality and blood feeding after exposure to insecticide-treated nets in experimental huts

		OR	*P* value	CI	OR	*P* value	CI
*Mortality*							
PermaNet 2.0			*CYP6P9a*			*CYP6P9b*	
Unfed	RR vs. SS	34.9	<0.0001	15.8–77.1	109.3	<0.0001	40.7–293.2
	RS vs. SS	19.9	<0.0001	9.7–40.9	34.7	<0.0001	14.6–82.5
	RR vs. RS	1.75	0.26	0.81–3.8	3.14	0.0058	1.4–6.9
	R vs. S	6.25	<0.0001	3.3–11.7	7.2	<0.0001	3.8–13.4
All samples	RR vs. SS	10.8	<0.0001	5.6–20.8	62.1	<0.0001	23.6–163.6
	RS vs. SS	5.3	<0.0001	2.8–9.8	21.9	<0.0001	8.7–55.1
	RR vs. RS	2.04	0.0002	1.1–3.7	2.8	0.0025	1.48–5.2
	R vs. S	3.17	0.02	1.78–5.65	4.7	<0.0001	2.6–8.7
*Blood feeding*							
PermaNet 2.0	RR vs. SS	1.75	0.19	0.82–3.7	1.29	0.54	0.58–2.9
	RR vs. RS	2.5	0.052	1.09–5.75	0.92	0.5	0.49–1.7
	RS vs. SS	0.7	0.67	0.28–1.7	1.68	1	0.79–3.5
	R vs. S	1.43	0.26	0.82–2.5	0.94	1	0.5–1.6
PermaNet 3.0	RR vs. SS	4.54	<0.0001	2.3–8.7	5.04	<0.0001	1.7–14.6
	RR vs. RS	2.6	0.0012	1.43–4.7	2.4	0.0085	1.31–4.39
	RS vs. SS	1.74	0.17	0.87–3.47	2.1	0.3	0.73–6.03
	R vs. S	2.14	0.18	1.17–3.19	4.1	<0.0001	2.2–7.5

Similarly, heterozygote mosquitoes (RS) survived exposure to PermaNet 2.0 significantly better than homozygote susceptible (SS) (OR = 34.7; CI = 14.6–82.5; *P* < 0.0001, Fisher’s exact test). The additive resistance conferred by each allele of *CYP6P9b* was shown by the fact that homozygote resistant mosquitoes (RR) could also survive better than heterozygotes (OR = 3.14; CI = 1.4–6.9: *P* = 0.0058, Fisher’s exact test). Overall, the strength of this association was further shown by the significant ability to survive exposure when possessing a single *CYP6P9b*-resistant allele (R) compared to the susceptible allele (S) (OR = 7.2; CI = 3.813.4; *P* < 0.0001, Fisher’s exact test) (Fig. 5b). A similar pattern of significant correlation between *CYP6P9b* genotypes and mortality was also observed when analysing all the dead and live samples regardless of blood feeding and exophilic status but with a lower OR value (Table 1).

The impact of the CYP6P9b_R was also assessed on the ability to blood feed against PBO-based net (PermaNet 3.0). A significant difference was observed in the distribution of *CYP6P9b* genotypes between blood-fed and unfed mosquitoes when exposed to PermaNet 3.0 (chi square = 28.8; *P* < 0.0001, chi-square). This resulted in a significant association between *CYP6P9b* genotypes and the ability to blood feed with homozygote resistant (RR) mosquitoes feeding significantly more than homozygote susceptible (SS) mosquitoes (OR = 5.04: CI = 1.7–14.6; *P* = 0.0001, Fisher’s exact test). This is also true for homozygote resistant (RR) when compared to heterozygotes (OR = 2.4: CI = 1.3–4.39; *P* = 0.0085, Fisher’s exact test). No significant difference was observed between heterozygotes and susceptible genotype (Table 1). However, *CYP6P9b* did not impact blood feeding when mosquitoes were exposed to PermaNet 2.0, and no difference was observed for the control net (Fig. 5c; Table 1). No significant association was observed for PermaNet 2.0 (Supplementary Fig. 4a) or in the control untreated net (Supplementary Fig. 4b).

### Combined effect of *CYP6P9b* and *6P9a* on LLIN efficacy

**Fig. 6:**
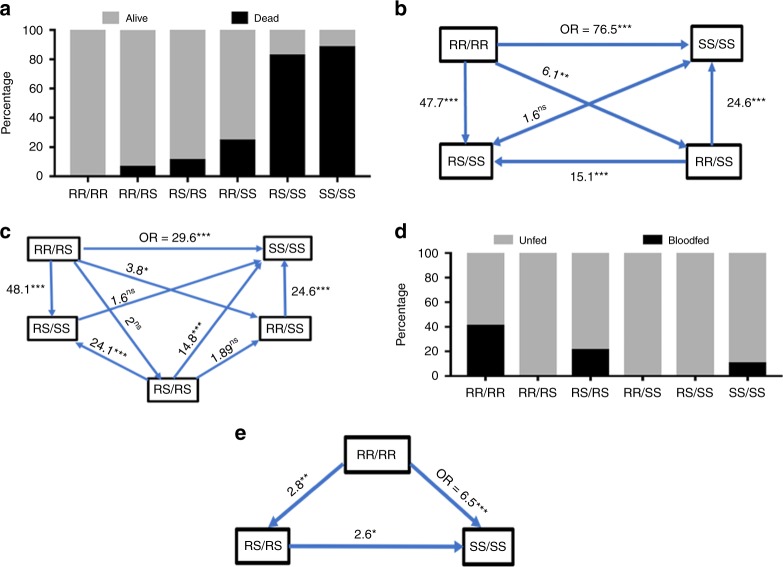
*CYP6P9b* combines with *CYP6P9a* to further reduce bed nets’ efficacy. **a** Distribution of the combined genotypes of both *CYP6P9a* and *CYP6P9b* showing that genotypes at both genotypes combined to additively increase the ability to survive after exposure to PermaNet 2.0. **b** Ability to survive exposure to PermaNet 2.0 (Odds ratio) of the double homozygote-resistant (RR/RR) genotypes of *CYP6P9a* and *CYP6P9b* compared to other genotypes supporting the additive resistance effect of both genes. Significance is shown by ^*^P < 0.05, ^**^P < 0.01, ^***^P < 0.001, as estimated using Fisher’s exact test. **c** Ability to survive exposure to PermaNet 2.0 (odds ratio) of the combined homozygote resistant and heterozygote (RR/RS) genotypes of *CYP6P9a* and *CYP6P9b* compared to other genotypes. **d** Distribution of the combined genotypes of both *CYP6P9a* and *CYP6P9b* after exposure to PermaNet 3.0 revealing an additive effect of both genes in increasing the ability to blood feed. **e** Comparison of blood feeding ability of combined genotypes of *CYP6P9a* and *CYP6P9b* showing a significantly higher ability (odds ratio) to blood feed for mosquitoes that double homozygote resistant (RR/RR). Source data are provided as a Source Data file

As *CYP6P9b* genotypes were shown to be independent from those of *CYP6P9a*, we next assessed how combinations of genotypes at both genes impact the efficacy of LLINs focusing on PermaNet 2.0 for mortality and PermaNet 3.0 for blood feeding. Analysis of the impact of combined genotypes on mortality with PermaNet 2.0 confirmed the independent segregation of genotypes at both genes with several combinations of genotypes observed including RR/RR, RR/RS, RS/RS, RS/SS and SS/SS (Fig. 6a). A comparison of the distribution of both sets of genotypes revealed that double homozygote resistant (RR/RR) mosquitoes at both genes had a far greater ability to survive exposure to PermaNet 2.0 than all other combinations. This shows that both genes act additively to confer a greater resistance to pyrethroids associated with a higher reduction of bed net efficacy when mosquitoes are double resistant. The additive resistance was further supported in that RR/RR mosquitoes had the greatest ability to survive exposure to PermaNet 2.0 when compared to double susceptible (SS/SS) (OR = 76.5; CI = 15.1–387.7) when considering all samples. There was even greater correlation if only considering unfed mosquitoes in the room (Fig. 6b). A significantly increased survival is also observed in RR/RR when compared to other combinations although with a lower odds ratio, such as against RS/SS (OR = 47.7; *P* < 0.0001, Fisher’s exact test), against RR/SS (OR = 6.1; *P* = 0.01, Fisher’s exact test) and against RS/RS (OR = 3.2; *P* = 0.042, Fisher’s exact test). No significant difference was observed between RR/RR and RR/RS (OR = 1.6; *P* = 0.82, Fisher’s exact test) (Fig. 6b). A similar trend is observed when comparing homozygote resistant mosquitoes to one gene and heterozygote to the other (RR/RS) against other combinations although at a lower strength (Fig. 6c). No significant difference is observed between RR/RS and RS/RS suggesting that having one resistance-associated allele of each gene is as good as having homozygote resistance for one gene.

Overall, a gradual decrease of impact is seen for genotypes RR/RR > RR/RS > RS/RS > RR/SS > RS/SS > SS/SS. Analysis of the combined genotype distribution for blood feeding (Fig. 6d) also revealed a significantly increased ability to blood feed for double homozygote-resistant mosquitoes when exposed to PermaNet 3.0 with the highest significance against double homozygote susceptible (OR = 6.5; *P* < 0.0001, Fisher’s exact test) (Fig. 6e; Supplementary Table 3). No significant association was observed for PermaNet 2.0 (Supplementary Fig. 4c) and in the control untreated net (Supplementary Fig. 4d).

## Discussion

The detection of the resistance markers associated with *CYP6P9b* over-expression described here provides a tool to monitor pyrethroid resistance in field populations of the major malaria vector *An. funestus*. In addition, it also allows the assessment of the interplay between P450 genes in their overall genetic variance to resistance as seen here between *CYP6P9a* and *CYP6P9b*. Overall, this study established key insights regarding the molecular basis of pyrethroid resistance in malaria vectors in Africa.

Genomic analyses performed in this study suggest differences in the genomic evolution of metabolic resistance to pyrethroids across Africa: mosquitoes from different geographic regions exhibited specific sets of genes and genetic variants associated with resistance. The comparative RNAseq-based transcription profiling between regions revealed key differences in the level of expression of major resistance genes, including *CYP6P9a* and *CYP6P9b* which are highly expressed in southern Africa. This difference in gene expression is in line with previous reports^11,13,17^. Other genes predominate in other regions with *CYP9K1* in Uganda, *CYP6P5* and *CYP325A* in Central Africa (Cameroon) and *CYP6P4a/b* in Ghana (West Africa). The resistance profile does not reflect the relatedness between populations^13^ suggesting each population independently evolves resistance mechanisms. This contrasting expression profile also supports previous observations^16^ and highlights the challenges in designing diagnostic tools for metabolic resistance in the face of such molecular plasticity of populations of malaria vectors. However, some of the differences observed between geographic regions could be the consequence of differences in genetic background between populations rather than just insecticide resistance. Further functional characterisation of these overexpressed genes both in vivo and in vitro^16^ will allow to confirm their role in resistance.

The detection of another P450 DNA-based marker in this study is a great progress in our efforts to detect and track the spread of metabolic resistance to insecticides in malaria vectors. The mutation associated with resistance here is located in the *cis*-regulatory region as established for *CYP6P9a*^13^ or for previous observations in the mosquito *Culex quinquefasciatus*^20^ and in *Drosophila*^21^. Similarly, the *CYP6P9b* AAC indel resistance mutation is associated with transcription factor binding sites notably to the insect ortholog of the mammalian transcript factor Nrf2, Cap ‘n’ Collar isoform-C (CncC) with Maf-S; these regulate transcription of detoxification genes in several insects^22^. This suggests that more attention should be paid to *cis*-regulatory regions to detect potential causative mutations that could drive resistance although this is not the unique mechanism as *trans*-acting regulatory loci^12^ or allelic variations of resistance genes^9^ may also be involved.

Because of the physical proximity of *CYP6P9b* to *CYP6P9a*, one could have assumed that both genes might be in complete linkage disequilibrium and any marker on one of the genes will fully explain resistance associated with the other. However, our data suggest otherwise; while we observed significant co-occurrence of resistance alleles in both genes, the genotypes at both genes can segregate independently and we observe increased pyrethroid resistance in mosquitoes with the combined genotype. The percentage of linkage between both genotypes varies significantly from nearly 100% in southern Africa to 71% in the East (Tanzania) and 51% in DRC. This suggests that both genotypes are not fully physically linked. The higher proportion of linked genotypes in southern Africa and Tanzania is possibly a result of a greater selection acting on this population as seen in southern Africa where proportion of identity is close to 100% and where high level of resistance has been reported^23,24^.

The independent segregation between *CYP6P9a* and *CYP6P9b* is also observed in the lab—the hybrid strain between the lab-resistant FUMOZ and lab-susceptible FANG exhibited significant independent genotypes for both genes at the F_4_ generation. Therefore, the availability of the CYP6P9b_R diagnostic assay developed here is an important tool that should increase our ability to detect and track the spread of resistance. Moreover, when combined with CYP6P9a_R, it could help to assess the risk of multi-gene driven high-resistance in this malaria vector. The current P450 diagnostic tools should allow control programmes to monitor both P450 alleles to track escalating pyrethroid resistance intensity in the field. This marker only applies to *An. funestus* and not to other malaria vectors including those of the *An. gambiae* complex. However, because *CYP6P3*, an ortholog of *CYP6P9a/b*, has also been shown to play an important role in insecticide resistance in *An. gambiae*, the results described here can serve as a model to detect similar markers in other malaria vectors, specifically of this species complex.

The experimental hut study performed here assessed the impact of *CYP6P9b* and revealed that *CYP6P9b* is associated with reduced efficacy of bed nets, particularly pyrethroid-only nets, independent of *CYP6P9a*^13^. However, the key message from this study is that a greater reduction of bed net efficacy is observed when the *CYP6P9b* resistant genotype combines with the *CYP6P9a* resistant genotype because double homozygote resistant mosquitoes survived exposure to pyrethroid-only nets much better than all other genotypes tested.

This shows the great risk that metabolic resistance poses to insecticide-based interventions as highlighted in the WHO global plan for insecticide-resistance management which states that if nothing is done pyrethroid resistance could lead to an increase burden of malaria in Africa^2^. This concern is further supported by a recent report from the 2018 World Malaria Report indicating that after nearly two decades of continued decline, malaria cases have significantly increased in 2017^25^. The spread of both CYP6P9b_R and CYP6P9a_R alleles poses a significant risk to the effectiveness of control programmes notably in southern Africa where both alleles are nearing fixation. The diagnostic tools designed here should help track their spread and help anticipate the management of such resistance. PBO-based nets (here PermaNet 3.0) are far more effective against such populations than pyrethroid-only nets, as also recently revealed in field trials in Tanzania^26^. Nevertheless, the high blood-feeding ability of resistant mosquitoes even against PBO-nets suggests that metabolic resistance affects the efficacy of such nets and calls for the development of other management options, for example nets that do not rely on pyrethroids. Our experimental hut trial study is based on a test with hybrid strains from two laboratory strains and the impact of the resistance alleles described here on the efficacy of LLINs in natural populations remains to be established, work that must be undertaken urgently.

In conclusion, this study used a comprehensive transcriptomic and targeted genomic approach to detect a P450-based resistance marker and designed a simple PCR assay to detect and track P450-mediated metabolic resistance to pyrethroid in the major malaria vector *An. funestus* in Africa. We show that the *CYP6P9b_R* resistance allele described here can combine with the previously detected *CYP6P9a_R*^19^ present on the same *rp1* QTL, conferring greater insecticide resistance in our bed nets test than either resistance genotype in isolation. Out of the tested populations, we detected this allele only in southern and eastern African mosquito populations. The greater reduction of efficacy of insecticide-treated nets observed in double-resistant mosquitoes is a major concern for the sustainability of insecticide-based interventions solely relying on pyrethroids. Therefore, PBO-based nets should be preferably deployed for greater impact while encouraging the development of alternative mosquito management options in malaria-endemic regions.

## Methods

### Collection and rearing of mosquitoes

The two *An. funestus* laboratory colonies used here are the FANG colony—a completely insecticide-susceptible colony originating from Angola—and the FUMOZ colony derived from southern Mozambique that is highly resistant to pyrethroids and carbamates^27^. Mosquito collection was performed to cover the four main regions in sub-Saharan regions: southern (Malawi, Chikwawa (16°1’ S, 34°47’ E) in 2014^28^), eastern (Uganda, Tororo (0°45’ N, 34°5’ E) 2014^29^), central (Cameroon, Mibellon (6°46′ N, 11°70′ E) in 2015^30^), and west (Ghana, Obuasi (5°56′ N, 1°37′ W) in 2014^31^) across the continental range of *An. funestus* as recently described^13^. Overall, blood-fed, gravid females were collected indoor in houses using electric aspirators between 06:00 AM-10.00AM in each location. Females were left to become fully gravid for 4 days and then introduced into 1.5 ml Eppendorf tube to lay eggs following the forced-egg laying method^32^. Larvae were reared to generate F_1_ adults and used for WHO bioassays as done by Weedall et al^13^. These populations are all resistant to pyrethroids and as well as to other insecticide classes^28–31^. Morphological and molecular identifications were performed to establish the species following Weedall et al.^13^.

### RNA extraction with library preparation and sequencing

The total RNA from three pools of ten female mosquitoes (alive after 1 h permethrin exposure) was extracted using the Arcturus PicoPure RNA isolation kit (Life Technologies, Carlsbad, CA, USA), according to the manufacturer’s instructions. This included a DNase treatment step. The total RNA was depleted for ribosomal RNA (rRNA) with Ribo-Zero low input kit for human/mouse/rat (Epicentre, Madison, WI, USA) using 100 ng of starting material. The Ribo-Zero mRNA-enriched material was used to prepare RNAseq libraries with the ScriptSeq v2 RNAseq library preparation kit (Epicentre) (15 cycles of PCR amplification). Agencourt AMPure XP beads (Beckman and Coulter, Beverly, MA, USA) were used to purify the libraries followed by a quantification using a Qubit fluorometer (Life Technologies). The size distribution was measured with a 2100 Bioanalyzer (Agilent, Santa Clara, CA, USA).

Pools of libraries (8/lane) were sequenced (2 × 125 bp paired-end sequencing) with v4 chemistry on a HiSeq 2500 (Illumina, San Diego, CA, USA). All sequence library preparation and sequencing were performed by the Centre for Genomic Research (CGR), University of Liverpool.

### Analysis of RNAseq data

RNAseq data were analysed as recently described when analysing the contribution of the *CYP6P9a* gene^13^, including the initial processing and quality assessment of the sequence data. The Subread aligner version 1.4.6^33^ was used to align the RNAseq R1/R2 read pairs to the reference sequence for which the annotation was improved using BLAST2GO version 4.0.7^34^.

FeatureCounts version 1.4.6^35^ was used to count the fragments mapped in the sense orientation to annotated *An. funestus* genes (automated predictions from gene set AfunF1.4, downloaded from VectorBase). EdgeR^36^ was used to analyse the differential gene expression with pairwise comparisons performed.

The differences in total tag counts among samples were corrected by calculating normalisation factors using the “TMM” (Trimmed Mean *M*-values) method in edgeR^36^ with default parameters. The *P* values associated with logFC were adjusted for multiple testing using the false-discovery rate (FDR) approach^37^. Differentially expressed genes were defined as those with an FDR-adjusted *P* value < 5% and> twofold absolute difference in expression level. Tag counts and the total CDS lengths were used to calculate the fragments per kilobase of gene sequence per million mapped reads.

Further analyses of the RNAseq data were performed using the Strand NGS software (Strand Life Sciences, version 3.0) following RNA alignment and RNA-seq analysis pipeline with standard parameters. Genes differentially expressed in each country and between different countries (generated from Venn diagrams) were detected using DESeq normalisation with a FC > 2. Multiple test correction used Benjamini–Hochberg method with a false discovery rate of 5% (adjusted *P* value < 0.05).

### Quantitative reverse transcriptase PCR

The expression patterns of the most differentially expressed detoxification genes between countries (Cameroon, Uganda, Malawi, Ghana and Benin) were validated using qRT-PCR. The primers are listed in Supplementary Table 4. Total RNA was extracted from three biological replicates from mosquitoes that survived 1 h exposure to permethrin in each country. One microgram was used as the template for cDNA synthesis using Superscript III (Invitrogen) with oligo-dT20 and RNase H according to the manufacturer’s instructions. The qRT-PCR amplification was performed following standard protocol^11,38^ after establishing the standard curves for each gene to assess PCR efficiency and quantitative differences between samples using serial dilution. The relative expression level and FC of each target gene was established for comparisons to Cameroon; these were calculated according to the 2^−ΔΔCT^ method incorporating the PCR efficiency^39^ after normalisation with the housekeeping genes ribosomal protein S7 (*RSP7*; AFUN007153) and actin 5C (AFUN006819).

### Analysis of *CYP6P9b* polymorphism patterns Africa-wide

Due to the high up-regulation of *CYP6P9b*, the polymorphism of its 5′UTR between *CYP6P9b* and *CYP6P5* was analysed by amplifying and sequencing a 1-kb fragment, following same protocol as for *CYP6P9a*^18^ in 15 mosquitoes in different regions of the continent. This included southern (Mozambique, Malawi and Zambia), central (DR Congo and Cameroon), eastern (Kenya and Uganda), and western Africa (Benin, Nigeria and Ghana) and in the two laboratory strains FANG and FUMOZ. The primers are presented in Supplementary Table 4. ClustalW^40^ was used to align the sequences, and the polymorphisms were identified in DnaSPv5.10^41^. The maximum likelihood tree was constructed using MEGA 7.0 with bootstrapping (500 replicates)^42^.

The 1 kb 5′UTR fragment of *CYP6P9b* was also amplified in mosquitoes collected in Mozambique before the scale up of bed nets (pre-intervention) in 2000 and in mosquitoes collected in 2016 in the same region after the deployment of long-lasting insecticidal nets in these countries. The PCR products were cloned and sequenced, and the sequencing data was analysed as described above while also constructing a haplotype network using TCS^43^.

### Promoter activity of 5′-flanking region sequences of *CYP6P9b*

To investigate the regulatory regions controlling *CYP6P9b*, the intergenic region between this gene and the preceding gene *CYP6P5* was used to design primers (6P9dplF and 6P9a/b) to amplify this region in the laboratory colonies FANG and FUMOZ. The PCR products were then cloned in to PJET1.2 (Thermo Scientific, St. Leon-Rot, Germany) and sequenced using the Pjet1.2 forward and reverse sequencing primers.

The promoter activities of the 5′-flanking region sequences were evaluated using a luciferase reporter assay. Primers (KpnI_6P9bF1.0 and HindIII_6P9a/b) were used to amplify the 5′-flanking region sequences for the resistant and the susceptible strains cloned in Pjet1.2 and then sub-cloned into the PGL3-basic (Promega, Madison, WI, USA) firefly luciferase vector between the Kpn1 and Hind111 restriction sites to form the recombinant promoter PGL3 for testing.

The recombinant constructs were transfected in *An. gambiae* cell line 4a-2 cell line (MRA-917 MR4, ATCC^®^ Manassas Virginia) maintained at 25 °C in Schneider’s *Drosophila* medium supplemented with 10% heat inactivated foetal calf serum and 1% penicillin/streptomycin. One day prior to transfection, about 5 × 10^5^ cells were plated in each well of 24-well plates and allowed to reach 60–70% confluence. At about 60% confluence, the constructs were introduced in the cells using Qiagen Effectene Transfection Reagent by co-transfecting 600 ng recombinant reporter constructs (*CYP6P9b* 5’flanking region in pGL3-Basic), LRIM promoter in pGL3 and pGL3 without promoter with 1 ng actin-Renilla internal control in 60 ml DNA condensation buffer, 4.8 ml enhancer and 6 ml Effectene in triplicates. Two independent replicates were carried out. Two days after incubation at 25 °C, the cells were washed with PBS and lysed in 100 ml passive lysis buffer (Promega); a luminometer (EG & G Berthold) was used to measure the luciferase activity, which was normalised to Renilla luciferase activity.

To localise the enhancer elements responsible for the up-regulation of *CYP6P9b* in the resistant as opposed to the susceptible populations, progressive 5′ deletion fragments of the 5′ flanking region from −978 to +97 (from the predicted transcription start site) was performed. Serially deleted fragments −630 to +97, −440 to +97, −181 to +97 and −128 to +97 were constructed using HindIII_6P9a/b common reverse primer, and each of the forward primer KpnI_6P9bF(1.0), KpnI_6P9bF(0.6), KpnI_6P9bF(0.4), KpnI_6P9bF(0.3) and KpnI_6P9bF(0.1) was tested to check if the deleted fragments −978 to −631, −630 to −441, −440 to −182 and −181 to −129 enhanced the promoter activity.

### Design of PCR-RFLP diagnostic for *CYP6P9b* genotyping

The SNP-RFLPing 2^44^ tool was used to design a PCR-RFLP that could discriminate between the *CYP6P9b* promoter of the resistant (FUMOZ) and the susceptible (FANG) strains. Primers 6P9brflp_0.5F and 6p9brflp_0.5R were designed to amplify a region in the promoter common both to FUMOZ and FANG. Each PCR reaction was performed in a 15-µL volume consisting of 1× buffer A, 25 mM MgCl_2_, 25 mM dNTPs, 10 mM of each primer 1U KAPA Taq polymerase (Kapa Biosystems, Boston, MA, USA). The following PCR amplification conditions were used initial denaturation step of 3 min at 95 °C, followed by 35 cycles of 30 s at 94 °C, 30 s at 58 °C, and 60 s at 72 °C with 10 min at 72 °C for final extension according to the KAPA kit instructions. Five microlitre of the PCR product was digested by adding 1 µL of CutSmart buffer 0.2 µL of *Tsp*45I restriction enzyme (New England Biolabs) and 3.8 µL of water. The mix was incubated at 65 °C for 2 h. The digested product was migrated on 2% agarose and stained with Midori Green Advance DNA Stain (Nippon genetics Europe GmbH) and visualised using a gel imaging system to confirm the product sizes; there was one band at 550 bp for the resistant (undigested) sample and two bands at 400 bp and 150 bp for the susceptible samples.

To validate the robustness of the PCR-RFLP to detect the pyrethroid resistance in the field population, the F_8_ progeny from a cross between highly resistant (FUMOZ) and highly susceptible (FANG) strains previously used for QTL mapping^14^ were genotyped and correlated with the resistance phenotype established using the odds ratio and Fisher’s exact test.

The pattern of geographical distribution of the resistant allele of *CYP6P9b* allele across the continent was assessed by genotyping the CYP6P9b_R in 30–50 field-collected females of *An. funestus* from several countries in Africa using the DNA-based diagnostic assay.

### Impact of CYP6P9b-on LLINs’ efficacy using hut trials

The study was performed in Mibellon (6°4′60″ N and 11°30′0″ E)—a village in the Adamawa region of Cameroon where 12 experimental huts have recently been built with concrete bricks following the specific design for experimental hut from the West Africa region^45^.

The study used a hybrid *An. funestus* strain generated from reciprocal crossing between the highly pyrethroid resistant strain FUMOZ-R (CYP6P9b_R) and the fully susceptible FANG strain (CYP6P9b_S)^9^. The F5 and F6 generations were used for the release experiments in the huts after the initial reciprocal crossing between the laboratory susceptible (FANG and resistant (FUMOZ) strains using 50 males and 50 females of both strains. The susceptibility of these strains to insecticides is reported by Weedall et al^13^ using pyrethroids (0.75% permethrin and 0.05% deltamethrin), DDT (4%) and the carbamate and bendiocarb (0.1%) according to WHO protocol^45^.

The following three treatments were compared in the experimental huts: (i) untreated polyethylene net; (ii) PermaNet 2.0® (deltamethrin incorporated into polyethylene net); and (iii) PermaNet 3.0^®^ (PBO + Deltamethrin incorporated into polyethylene net). To simulate a worn net, six 4 cm × 4 cm holes were made on each net according to WHO guidelines. The hybrid FANG/FUMOZ strain was released in each hut for 6 nights (80 mosquitoes per hut).

Three adult volunteers were recruited from the Mibellon village to sleep under the nets and collect mosquitoes in the morning. Each volunteer provided a written consent to participate in this study and were also given chemoprophylaxis during the trial. Ethical approval was obtained from the National Ethic Committee of the Ministry of the Health in Cameroon.

Mosquito collection was performed in the morning by each volunteer in their respective room using glass tubes. Mosquitoes were collected in three compartments including the main room (the walls, floor and the hut ceiling), inside of each net and in the exit traps (veranda). A single bag was used for each compartment to avoid the risk of mixing samples from different areas. A sugar solution was provided to mosquitoes found alive and they were kept in paper cups for 24 h to assess the delayed mortality. The status of collected mosquitoes was recorded in observation sheets either as alive/blood fed, dead/blood fed, alive/unfed and dead/unfed.

An untreated net was used as control to estimate the effect of each treatment by assessing several parameters including the induced exophily (the proportion of mosquitoes exiting the room early through the exit traps), the mortality rates (which provides an indication of the potential mass killing effect of the bed nets) and the blood feeding rate (which estimates the level of personal protection).

A direct comparison to the untreated control net was used to establish the effect of both treated nets. A logistic regression model using the Wald statistic that follows a chi-squared distribution (with d*f* = 1) was used to assess the statistical significance of the difference.

The CYP6P9b_R resistance allele was genotyped in a subset of each treatment to determine the impact of the *CYP6P9b-*mediated metabolic resistance to pyrethroids on the effectiveness of the bed nets. The following were included: the dead, alive, blood-fed, and unfed mosquitoes in the veranda, in the net and in the room. The association between the mutation and the performance of each net was assessed using the Vassar stats (http://vassarstats.net/) with a 2 × 2 contingency table for the odds ratio calculation based on Fisher’s exact probability test.

### Reporting summary

Further information on research design is available in the Nature Research Reporting Summary linked to this article.

## Supplementary information

Supplementary Information

Reporting Summary

Description of Additional Supplementary Files

Supplementary Data 1

Source Data

## Data Availability

*RNAseq*: PRJEB24351 and PRJEB10294; *CYP6P9b sequences*: GenBank MK457459-MK457689. Data analysed in this study are available on public repositories or available within the article and its Supplementary Information files. Further details are available from the authors upon request.

## Supplementary information

**Supplementary information** is avaliable for this paper at 10.1038/s41467-019-12686-5.
