# Supplementary material for: Cis-regulatory CYP6P9b P450 variants associated with loss of insecticide-treated bed net efficacy against Anopheles funestus
Source: Nat Commun. 2019 Oct 11;10:4652. doi: 10.1038/s41467-019-12686-5 (PMC6789023; doi:10.1038/s41467-019-12686-5)
Supplement: Supplementary file 1 — Supplementary Information [file 41467_2019_12686_MOESM1_ESM.pdf]

Supplementary Information

Cis-regulatory *CYP6P9b* variants associated with loss of insecticide-treated bed net efficacy against *Anopheles funestus*

Mugenzi et al

Supplementary Figures

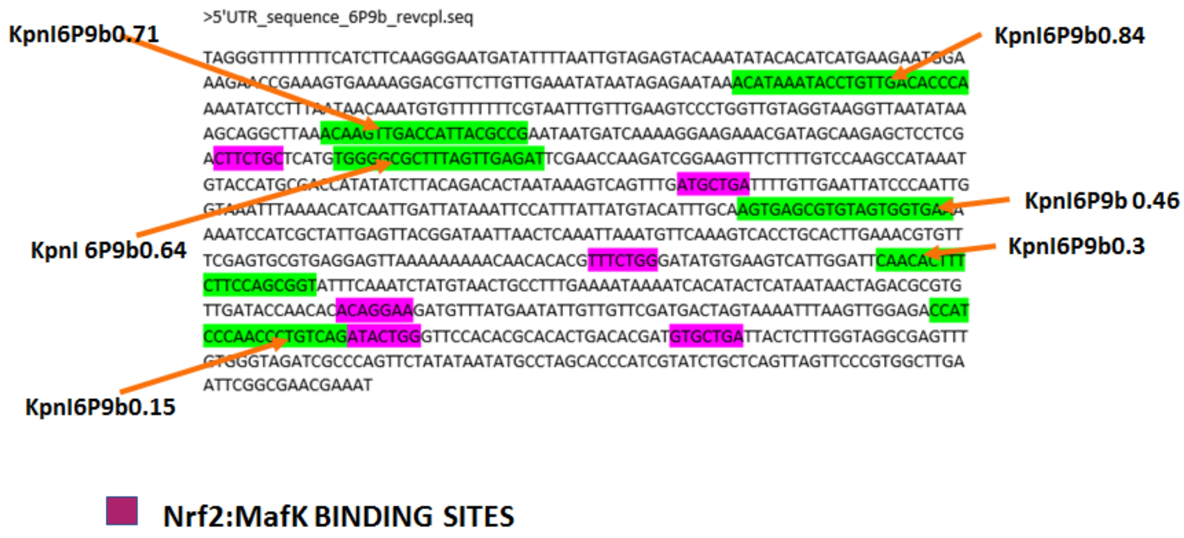

Supplementary Figure 1: Sequence of the intergenic region between *CYP6P9b* and *CYP6P5* harboring the regulatory element driving the upregulation of *CYP6P9b.*

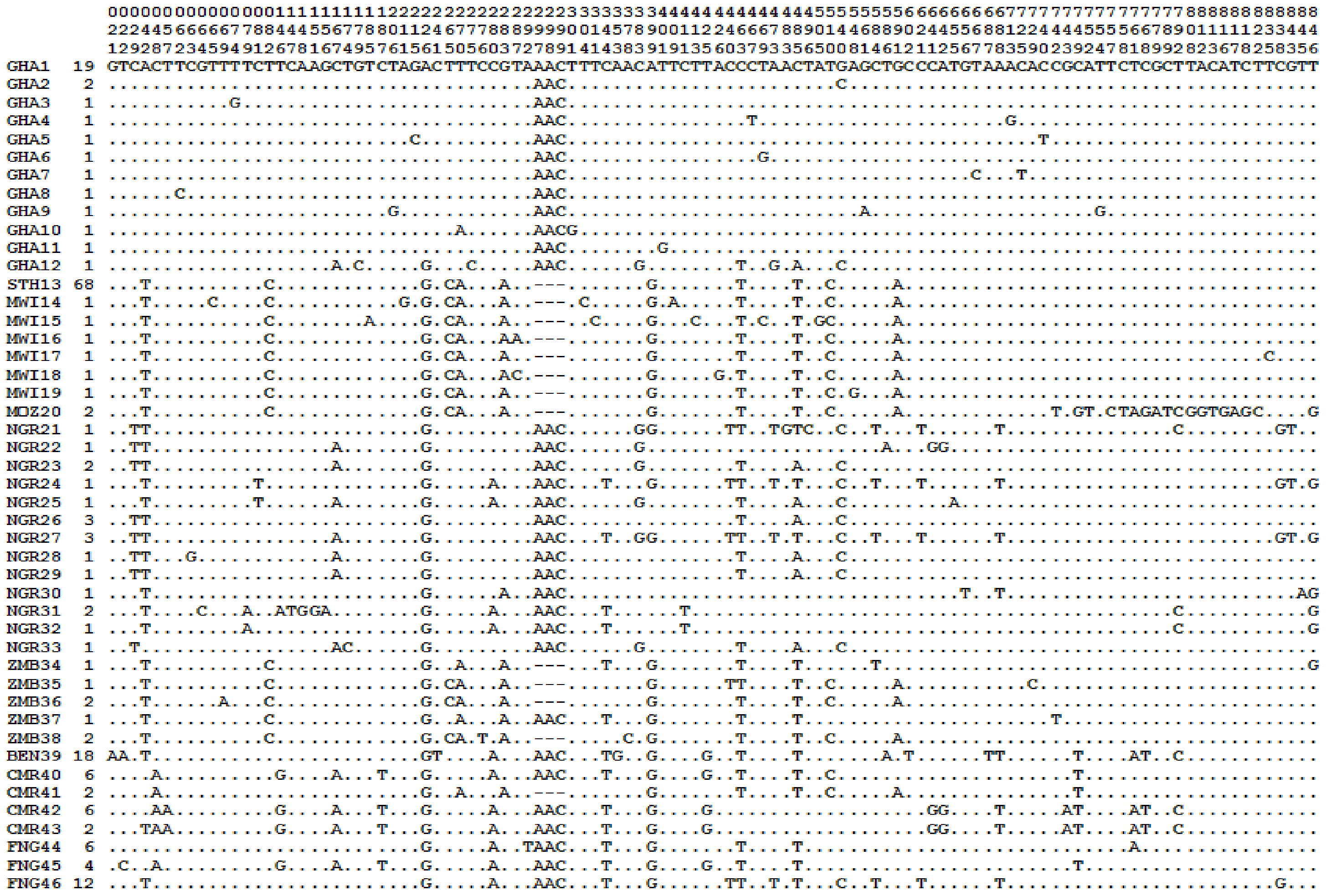

Supplementary Figure 2: Polymorphic sites and haplotypes of the *CYP6P9b* promoter region across Africa. Haplotypes are labeled with prefixes from the country where they are predominant. CMR, Cameroon, GHA is Ghana, BEN is Benin, MOZ is Mozambique, STH is Southern Africa, MWI is Malawi, ZMB is Zambia, FNG is FANG, NGR is Nigeria

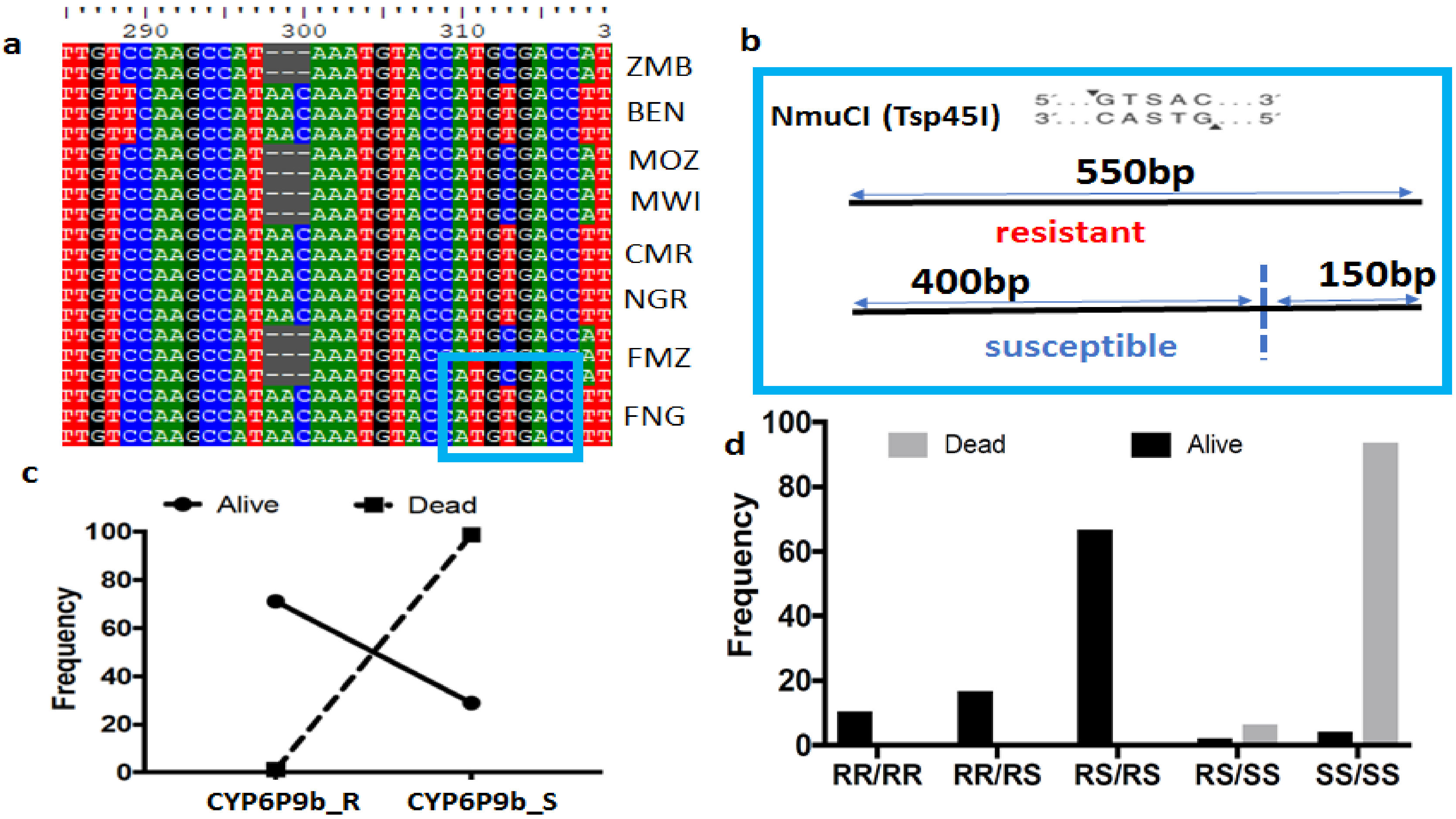

Supplementary Figure 3: Association between the *CYP6P9b* resistance marker and pyrethroid resistance: (a) Schematic alignment of sequences across Africa showing fixed variations associated with CYP6P9b-mediated pyrethroid resistance including the AAC insert only found in resistant mosquitoes and the C/T variant tightly linked to the resistant haplotype and generating a cut site for the NmuCl restriction enzyme.

(b) Schematic representation of the restriction digestion of the *CYP6P9b* fragment to differentiate between resistant and susceptible mosquitoes. (c) Frequency of *CYP6P9b* alleles between resistant (alive) and susceptible (dead) mosquitoes. (d) Frequency of various combinations of genotypes between *CYP6P9a* and *CYP6P9b* in F8 hybrid strain (FUMOZ_X_Fang) suggesting an independent segregation of genotypes of both genes in this hybrid strain.

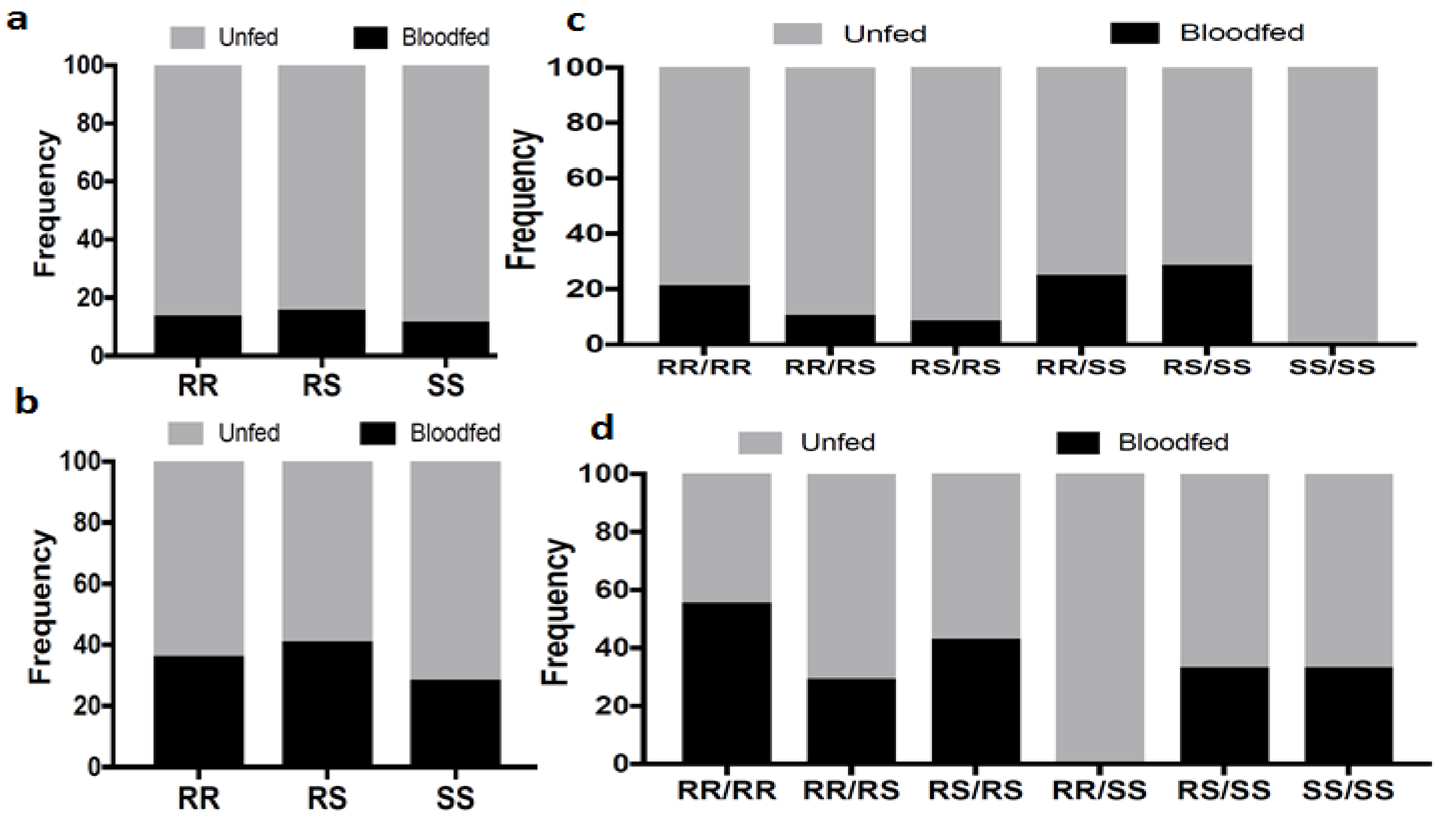

Supplementary Figure 4: impact of the CYP6P9b-mediated pyrethroid resistance on blood feeding after exposure to insecticide-treated nets: (a) Distribution of *CYP6P9b* genotypes between blood fed and unfed mosquitoes after exposure to the pyrethroid-only net PermaNet 2.0 showing no effect of *CYP6P9b* for this net. (b) is for the untreated nets showing no correlation between *CYP6P9b* genotypes and blood feeding success. (c) Distribution of the combined genotypes of both *CYP6P9a* and *CYP6P9b* after exposure to PermaNet 2.0 revealing no combined

effect of both genes in increasing ability to blood feed for this net. (d) is for the untreated nets with no association between combined genotypes and ability to blood feed.

Supplementary Tables

Supplementary Table 1: Detoxification genes differentially expressed in Malawi between different comparisons at FDR<0.05 and fold change (FC) >2.

Gene ID	R-C	C-S	R-S	Description
AFUN011266	2.1	2.3	4.8	UDP-glucuronosyltransferase 3A1
AFUN004798		2.6	3.9	ATP-binding cassette sub-family G member 2-like
AFUN001382		4.0	2.4	Cytochrome P450, CYP9J11
AFUN001383		3.3	3.1	Cytochrome P450, CYP9J11
AFUN006135		2.0	2.6	Cytochrome P450, CYP4C36
AFUN015785		2.2	2.2	Cytochrome P450, CYP6AA2
AFUN015792		57.1	60.1	Cytochrome P450, CYP6P9a
AFUN015889		18.5	23.7	Cytochrome P450, CYP6P9b
AFUN015795		4.1	2.1	Cytochrome P450, CYP6M7
AFUN015891		4.9	3.7	cytochrome P450 3A30-like
AFUN008239		2.1	3.8	Sulfotransferase 1B1
AFUN015907		2.4	2.2	Cytochrome P450, CYP305A3
AFUN015839		5.5	3.3	glutathione S-transferase, GSTD3
AFUN015807		2.8	2.7	glutathione S-transferase, GSTE1
AFUN015809		2.1	2.3	glutathione S-transferase, GSTE2
AFUN015811		2.1	2.2	glutathione S-transferase, GSTE5
AFUN016008		3.1	4.1	glutathione S-transferase, GSTE6
AFUN015801		2.4	2.5	Cytochrome P450, CYP6P2
AFUN010918		2.3	3.2	Cytochrome P450, CYP6N1
AFUN004002	2.5		2.4	argininosuccinate_lyase
AFUN000422	2.1			Carboxylesterase
AFUN006858	2.1			cytochrome P450, CYP306A1
AFUN015808	2.3			glutathione S-transferase, GSTE3
AFUN008941	2.6			ATP-binding cassette sub-family C member 1
AFUN008942	2.2			ATP-binding cassette sub-family C member 1
AFUN007549		2.1		Cytochrome P450, CYP9K1

R-C, Permethrin-resistant vs. control mosquitoes; C-S, control vs and the susceptible FANG strain mosquitoes; R-S, Permethrin-resistant and the susceptible FANG strain mosquitoes

Supplementary Table 2: Population genetic parameters of the 1kb fragment upstream of *CYP6P9b*

**Samples**	**N**	**S**	**h**	**hd**	**π**	**k**	**D**	**D***
Malawi	25	13	7	0.43	0.0013	1.1	-2.42**	-3.96**
Mozambique	20	18	2	0.189	0.004	3.4	-1.23	1.56**
Zambia	30	13	6	0.414	0.0015	1.36	-1.93*	-0.70ns
Ghana	31	27	12	0.63	0.002	1.85	-2.58***	-4.6**
Nigeria	19	40	13	0.95	0.015	11.9	-0.015^{ns}	0.09ns
Cameroon	16	21	4	0.73	0.0095	8.2	1.19^{ns}	1.5*
Benin	18	0	1	0	/	/	/	/
FUMOZ-R	8	0	1	0	/	/	/	/
FANG	22	21	3	0.62	0.0	9.14	2.2*	1.6*
Total Africa	189	105	46	0.846	0.015	12.5	-1.12^{ns}	-2.72*
Mozambique Pre-bednet	22	18	6	0.797	0.011	7.3	1.74^{ns}	1.56*
Mozambique Post-bednet	20	18	2	0.189	0.0053	3.4	-1.2^{ns}	1.56*
Total all	42	37	8	0.767	0.0157	10.1	0.48^{ns}	1.86*

N= number of sequences (2n); S, number of polymorphic sites; h, number of haplotypes; hd, haplotype diversity; π, nucleotide diversity (k= mean number of nucleotide differences); D and D* Tajima's and Fu and Li's statistics; ns, not significant; * significant P<0.05.

Supplementary Table 3: Additive impact of combined genotypes of *CYP6P9a* and *CYP6P9b* on the efficacy of insecticide-treated nets

	OR	P value	CI
**Mortality with PermaNet 2.0**			
RR/RR vs SS/SS	76.5	<0.00001	15.1-387.7
RR/RR vs RS/SS	47.7	<0.00001	12.6-181.01
RR/RR vs RR/SS	6.1	0.01	1.2-29.2
RR/RR vs RR/RS	1.6	0.82	0.5-5.07
RR/RR vs RS/RS	3.2	0.042	1.1-9.4
RR/RS vs SS/SS	29.6	<0.00001	6.3-139.4
RR/RS vs RS/SS	48.1	<0.00001	10.5-221.5
RR/RS vs RR/SS	3.8	0.011	0.9-16.02
RR/RS vs RS/RS	2	0.15	0.8-4.8
RS/RS vs SS/SS	14.8	<0.00001	3.3-65.7
RS/RS vs RS/SS	24.1	<0.00001	5.5-104.4
RS/RS vs RR/SS	1.89	1	0.48-7.5
RR/SS vs SS/SS	7.8	0.013	1.2-51.2
RR/SS vs RS/SS	12.7	<0.0001	1.9-81.6
RS/SS vs SS/SS	1.6	0.68	0.3-8.3
**Blood feeding with PermaNet 3.0**			
RR/RR vs SS/SS	6.5	0.0001	2.2-19.1
RR/RR vs RS/RS	2.86	<0.00001	1.5-5.4
RS/RS vs SS/SS	2.26	0.028	0.78-6.5
RS/RS vs SS/SS	2.24	0.06	1.0-4.9

Supplementary Table 4: List of primers used in this study

Gene	Forward primer	Reverse primer	Expected size (bp)
**Primers used for qRT-PCR**			
**CYP6P9a**	CAGCGCGTACACCAGATTGTGTAA	TCACAATTTTTCCACCTTCAAGTAATTACCCGC	92
**CYP6P9b**	CAGCGCGTACACCAGATTGTGTAA	TTACACCTTTTCTACCTTCAAGTAATTACCCGC	97
**CYP6M7**	CCA GAT ACT GAA AGA GAG CCT TCG	CAAGCACTGTCTTCGTACCG	102
**CYP6AA1**	CATCTGGCTGAATGGCACTA	TCAACAATGCCATCAAATCG	109
**CYP9J11**	CAAATTTAAAGAGTGCGCTAGG	GTAGATGGTGCCAAGGATGG	115
**CYP6Z1**	GGATTTCCGATGAGGATTGA	GCAGCGTACTTGATTTACGG	78
**CYP9K1**	AGGGCTTCTGGATACGGTTC	CGTACGGTTCGGTTTTGATT	103
**CYP6P4A**	AACTCGTATTCGACCCCAAA	CGTTTCCATGGAATTACATTTTCTG	146
**CYP6M4**	CACTATTCTCTCGCCGAAGG	CAAAGGATCCGCCATTCTAC	119
**CYP6N1**	GAAGCATTTCCGTTTTACGC	GGTGGCTTTATAGCTCGTT	138
**Ald Oxi (AFUN004380)**	GACTGGCAGACGATTGGATT	TGTAATCCAGCAACGGTGTC	134
**GSTe2**	GTTTGAAGCAGTTGCCATACTACGAGG	TCAAGCTTTAGCATTTTCCTCCTTTTTGGC	101
**Carb2514**	CAAACATGGCAATCCAACAC	CAAACTCACGTAACTCAATCATTTG	125
**CYP6P5**	ACGTAATCAACGAAACGCTTCGTA	TCGGTATCTGCACGATTGTT	126
**CYP325A**	GGATACCGATACGGCATGTT	TGCGTATTTGATGCCCTACA	148
**RSP7**	GTGTTCGGTTCCAAGGTGAT	TCCGAGTTCATTTCCAGCTC	98
**ACTIN**	TTAAACCCAAAAGCCAATCG	ACCGGATGCATACAGTGACA	111
**CYP6P9b_5'UTR F/R**	CCCCCACAGGTGGTAACTATCTGAA	TACACTGCCGACACTACGAAG	1073
KpnI_6P9bF(1.0)/ HindIII_6P9a/b	CGGGGTACCCCGCCCCCACAGGTGGTAACTATCTGAA	CCCAAGCTTGGGTACACTGCCGACACTACGAAG	

	Serial deletion of 5’UTR region of CYP6P9b		
KpnI_6P9bF(0.6):	CGGGGTACCCCGTGGGGCGCTTTAGTTG AGAT		
KpnI_6P9bF(0.4):	CGGGGTACCCCGAGTGAGCGTGTAGTG GTGAA		
KpnI_6P9bF(0.3)	CGGGGTACCCCGCAACACTTTCTTCCAG CGGT		
KpnI_6P9bF(0.1)	CGGGGTACCCCGCCATCCCAACCCTGTC AGAT		
6P9brflp_0.5F/6P9 brflp_0.5R	CCCCCACAGGTGGTAACTATCTGAA	TTATCCGTAACTCAATAGCGATG	550

Supplementary Notes

1-Africa-wide transcription analysis

Anopheles funestus populations of some of the regions have similar patterns such as West and Central Africa—these both exhibit a greater up-regulation of detoxification genes, *GSTe2* and *GSTe4,* than eastern and southern Africa although with greater FC in Ghana than Cameroon.

Despite the greatest geographical difference, western and southern Africa shared the most similarities with significant over-expression of several key genes such as *CYP6P9a* and *CYP6P9b* and *CYP6P4a/b* when compared to Central and East Africa although with differences in FC. These two regions also share the up-regulation of other detoxification genes such as the P450s *CYP6N1* and *CYP6P2* when compared to Central and East Africa.

The P450 *CYP6P5* was consistently upregulated in all other regions when compared to southern Africa with FC of 6.9, 4.4 and 6.3, respectively, in Cameroon, Uganda and Ghana. Overall, Malawi transcription pattern greatly differs from Uganda than other pairwise comparison of populations when analysing the list of genes differentially expressed from a Venn diagram of overlapping genes. This difference between both regions is characterized by the up-regulation of several detoxification gene families in Malawi compared to Uganda (Supplementary Table 1). This could be due to the multiple and high resistance observed in Malawi [18]. This could also be due to the complete absence of carbamate resistance in Uganda and not necessarily because of a greater genetic differentiation between the two regions since Uganda has been shown to be intermediate between southern and central or west populations [19,20].

2-Triangular analysis of gene expression in Malawi between R-C/R-S/C-S for Malawi

By comparing the resistant (R), unexposed control (C) and susceptible (S) mosquito samples only a single detoxification gene, the UDP-glucuronosyltransferase AFUN011266 was commonly over-expressed in the three comparisons (R-C, R-S and C-S) with highest FC in R-S (4.8) (Table S2). However, when analysing the set of genes commonly over-expressed between R-S and C-S, the duplicated P450 *CYP6P9a* and *CYP6P9b* were by far the most over-expressed detoxification genes with a slightly higher level in R-S compared to C-S (FC60.1 vs 57.1 for *CYP6P9a*; FC23.7 vs 18.5 for *CYP6P9b*). This common over-expression in both R-S and C-S comparisons supports that *CYP6P9a* and *CYP6P9b* are constitutively over-expressed in this population and supports that *CYP6P9b* is also playing a key role in the pyrethroid resistance. Analysis of the set of genes commonly over-expressed between R-S and C-S also detected several other cytochrome P450 genes located in previously described quantitative Trait Loci (QTLs) associated with pyrethroid regions. These include *CYP6P4a, CYP6P2* and *CYP6AA2* in the *rp1* (resistance to pyrethroid 1), *CYP6M7* and *CYP6N1* in *rp2* [1] and CYP9J11 in *rp3* [2]. However, all these P450s had an over-expression fold-change <5 further highlighting that *CYP6P9a* and *CYP6P9b* could be explaining most of the genetic variance of pyrethroid resistance in this region. Similarly, a set of glutathione S-transferases from epsilon class were commonly over-expressed in R-S and C-S including *GSTe1, GSTe2, GSTe5* and *GSTe6* in addition of *GSTD3* with FC<6 consistent with previous reports [3-5].

Only one commonly over-expressed gene was detected between R-C and R-S, the argininosuccinate lyase (AFUN004002) suggesting that this could be induced by exposure to insecticide. Other genes were only over-expressed in one comparison such as the carboxylesterase (AFUN000422), the glutathione S-Transferase *GSTe3* and the ABC transporter (ABCC1) in R-S whereas *CYP9K1* was over-expressed in C-S.

3-qRT-PCR validation of RNAseq expression patterns

Quantitative real-time PCR (qRT-PCR) was performed to confirm the differential expression observed between countries for key detoxification genes including twelve P450 genes, one glutathione S-transferase gene (*GSTe2*), a carboxlesterase gene (AFUN002514) and one aldehyde oxidase gene (AFUN004380). Overall, the qRT-PCR results confirmed the over-expression patterns observed by RNAseq between the countries. For a comparison purpose, the expression of all countries was compared to that of Cameroon (Figure 1) revealing set of genes over-expressed in other countries (Positive FC) or rather upregulated in Cameroon. This analysis confirmed that the P450s *CYP6P5* and *CYP325A* were both significantly over-expressed in Cameroon compared to other locations as well as the carboxylesterase AFUN002514 as suggested by RNAseq. It also confirms that other genes such as *CYP6P9a* and *CYP6P9b* were more up-regulated in Malawi, whereas *CYP9K1* was more over-expressed in Uganda and *CYP6P4a* in Ghana. Overall, qRT-PCR expression patterns support the differences observed in the main gene driving pyrethroid resistance in different African regions.

4-Genetic polymorphisms associated with CYP6P9b-mediated resistance

We amplified the whole 1kb intergenic region between *CYP6P9b* and *CYP6P5*. A 1kb PCR product was obtained in southern Africa, also in the lab resistant FUMOZ strain and the susceptible FANG. However, in East (Uganda) and Central Africa (Cameroon), some mosquitoes did not provide a band despite optimisation of the PCR suggesting potential structural variations or polymorphisms in this region preventing the amplification.

Supplementary references

1 Irving, H., Riveron, J. M., Ibrahim, S. S., Lobo, N. F. & Wondji, C. S. Positional cloning of rp2 QTL associates the P450 genes CYP6Z1, CYP6Z3 and CYP6M7 with pyrethroid resistance in the malaria vector Anopheles funestus. *Heredity (Edinb)* **109**, 383-392, doi:10.1038/hdy.2012.53 (2012).

2 Riveron, J. M. *et al.* Genome-Wide Transcription and Functional Analyses Reveal Heterogeneous Molecular Mechanisms Driving Pyrethroids Resistance in the Major Malaria Vector Anopheles funestus Across Africa. *G3 (Bethesda)* **7**, 1819-1832, doi:10.1534/g3.117.040147 (2017).

3 Barnes, K. G. *et al.* Restriction to gene flow is associated with changes in the molecular basis of pyrethroid resistance in the malaria vector Anopheles funestus. *Proc Natl Acad Sci U S A* **114**, 286-291, doi:10.1073/pnas.1615458114 (2017).

4 Riveron, J. M. *et al.* Directionally selected cytochrome P450 alleles are driving the spread of pyrethroid resistance in the major malaria vector Anopheles funestus. *Proc Natl Acad Sci U S A* **110**, 252-257, doi:10.1073/pnas.1216705110 (2013).

5 Weedall, G. M. *et al.* A single cytochrome P450 allele conferring pyrethroid resistance in a major African malaria vector is reducing bednet efficacy. *Science Translational Medicine* (In Press).
