# Supplementary material for: Cis-regulatory CYP6P9b P450 variants associated with loss of insecticide-treated bed net efficacy against Anopheles funestus
Source: Nat Commun. 2019 Oct 11;10:4652. doi: 10.1038/s41467-019-12686-5 (PMC6789023; doi:10.1038/s41467-019-12686-5)
Supplement: Supplementary file 2 — Reporting Summary [file 41467_2019_12686_MOESM2_ESM.pdf]

nature research

Corresponding author(s): Charles Wondji

Last updated by author(s): Aug 7, 2019

Reporting Summary

Nature Research wishes to improve the reproducibility of the work that we publish. This form provides structure for consistency and transparency in reporting. For further information on Nature Research policies, see Authors & Referees and the Editorial Policy Checklist.

Statistics

For all statistical analyses, confirm that the following items are present in the figure legend, table legend, main text, or Methods section.

n/a	Confirmed	
☐	☒	The exact sample size (*n*) for each experimental group/condition, given as a discrete number and unit of measurement
☐	☒	A statement on whether measurements were taken from distinct samples or whether the same sample was measured repeatedly
☐	☒	The statistical test(s) used AND whether they are one- or two-sided<br>*Only common tests should be described solely by name; describe more complex techniques in the Methods section.*
☒	☐	A description of all covariates tested
☐	☒	A description of any assumptions or corrections, such as tests of normality and adjustment for multiple comparisons
☐	☒	A full description of the statistical parameters including central tendency (e.g. means) or other basic estimates (e.g. regression coefficient) AND variation (e.g. standard deviation) or associated estimates of uncertainty (e.g. confidence intervals)
☒	☐	For null hypothesis testing, the test statistic (e.g. *F*, *t*, *r*) with confidence intervals, effect sizes, degrees of freedom and *P* value noted<br>*Give P values as exact values whenever suitable.*
☒	☐	For Bayesian analysis, information on the choice of priors and Markov chain Monte Carlo settings
☒	☐	For hierarchical and complex designs, identification of the appropriate level for tests and full reporting of outcomes
☒	☐	Estimates of effect sizes (e.g. Cohen's *d*, Pearson's *r*), indicating how they were calculated

Our web collection on statistics for biologists contains articles on many of the points above.

Software and code

Policy information about availability of computer code

Data collection	None
Data analysis	Strand NGS 3.2<br>SNP-RFLPing 2<br>DnaSPv5.10<br>MEGA 7.0<br>TCS 2.1

For manuscripts utilizing custom algorithms or software that are central to the research but not yet described in published literature, software must be made available to editors/reviewers. We strongly encourage code deposition in a community repository (e.g. GitHub). See the Nature Research guidelines for submitting code & software for further information.

Data

Policy information about availability of data

All manuscripts must include a data availability statement. This statement should provide the following information, where applicable:

- Accession codes, unique identifiers, or web links for publicly available datasets
- A list of figures that have associated raw data
- A description of any restrictions on data availability

RNAseq: PRJEB24351, PRJEB10294; CYP6P9b sequences: GenBank MK457459-MK457689. Data analysed in this study are available on public repositories or available within the article and its Supplementary Information files. Further details are available from the authors upon request.

Field-specific reporting

Please select the one below that is the best fit for your research. If you are not sure, read the appropriate sections before making your selection.

☒ Life sciences ☐ Behavioural & social sciences ☐ Ecological, evolutionary & environmental sciences

For a reference copy of the document with all sections, see nature.com/documents/nr-reporting-summary-flat.pdf

Life sciences study design

All studies must disclose on these points even when the disclosure is negative.

Sample size	Transcriptome profiling of An. funestus populations from four African regions---southern (Malawi), East (Uganda), West (Ghana), Central (Cameroon)--- were analysed to detect key candidate resistance genes. We used three replicates of total RNA from each location. This number was determined based on previous studies showing that this is enough to detect differentially expressed genes associated with resistance (Riveron et al 2013, PNAS, Weedall et al 2015, BMC Genomics). Same for done for qRT-PCR on three different total RNA samples. Number of sleepers for experimental huts was guided by the number of bed nets to test.
Data exclusions	No data exclusion
Replication	Data were replicated with minimum of three replicates for RNAseq, qRT-PCR, and for experimental hut trials
Randomization	This is not relevant for our study as we performed RNAseq from 3 replicates of mosquitoes alive after exposure to insecticides. For experimental huts we rotate sleepers per hut so no need of randomization
Blinding	not relevant

Reporting for specific materials, systems and methods

We require information from authors about some types of materials, experimental systems and methods used in many studies. Here, indicate whether each material, system or method listed is relevant to your study. If you are not sure if a list item applies to your research, read the appropriate section before selecting a response.

Materials & experimental systems

n/a	Involved in the study	
☒	☐	Antibodies
☒	☐	Eukaryotic cell lines
☒	☐	Palaeontology
☒	☐	Animals and other organisms
☐	☒	Human research participants
☒	☐	Clinical data

Methods

n/a	Involved in the study	
☒	☐	ChIP-seq
☒	☐	Flow cytometry
☒	☐	MRI-based neuroimaging

Human research participants

Policy information about studies involving human research participants

Population characteristics	Men aged 20-30 were selected from the village with no sign of any sickness.
Recruitment	Three adult volunteers were recruited from the Mibellon village to sleep under the nets and collect mosquitoes in the morning. They were provided a written consent form and were also given chemoprophylaxis during the trial
Ethics oversight	National Ethic Committee of the Ministry of the Health in Cameroon

Note that full information on the approval of the study protocol must also be provided in the manuscript.
