# Supplementary material for: Cis-regulatory CYP6P9b P450 variants associated with loss of insecticide-treated bed net efficacy against Anopheles funestus
Source: Nat Commun. 2019 Oct 11;10:4652. doi: 10.1038/s41467-019-12686-5 (PMC6789023; doi:10.1038/s41467-019-12686-5)
Supplement: Supplementary file 3 — Description of Additional Supplementary Files [file 41467_2019_12686_MOESM3_ESM.pdf]

## Description of Additional Supplementary Files

**Supplementary Data 1:** List of detoxification-associated genes differentially expressed when each country is directly compared to others using a Venn-diagram at FDR<0.05 and fold change (FC) >2
