# Supplementary material for: Cis-regulatory CYP6P9b P450 variants associated with loss of insecticide-treated bed net efficacy against Anopheles funestus
Source: Nat Commun. 2019 Oct 11;10:4652. doi: 10.1038/s41467-019-12686-5 (PMC6789023; doi:10.1038/s41467-019-12686-5)
Supplement: Supplementary file 4 — Supplementary Data 1 [file 41467_2019_12686_MOESM4_ESM.pdf]

Supplementary Data 1: List of detoxification-associated genes differentially expressed when each country is directly compared to others using a Venn-diagram at FDR<0.05 and fold change (FC) >2

Gene ID	Fold-change difference between countries													Mean read number				
	**MWI/CMR**	**MWI/UG**	**MWI/GHA**	**CMR/MWI**	**CMR/UGA**	**CMR/GHA**	**UGA/CMR**	**UGA/MAL**	**UGA/GHA**	**GHA/CMR**	**GHA/MWI**	**GHA/UGA**	**Description**	**FNG**	**CMR**	**GHA**	**MWI**	**UGA**
AFUN015792	28.3	22.7	9.5							2.9		2.4	Cytochrome P450, CYP6P9a	715.7	1379	5077	63009	2189
AFUN015889	5.5	3.4	3.5										Cytochrome P450, CYP6P9b	668.3	2628	5152	23226	5356
AFUN006981	3.9						2.2			2.3			cuticular_protein	122.6	15.1	42.2	96.1	41.5
AFUN015841	3.8	2.9								2.4			glutathione S-transferase, GSTD4	53.2	27.1	78.3	166.6	45
AFUN002055	3.8		4										lipase member H-like	20.4	7.2	8.6	44.4	11.9
AFUN015891	3.5						2.5			22.7	6.5	9	cytochrome P450, CYP6P4-like	111.4	105.8	2979	599.4	340.4
AFUN015890	3.4	2.8								25.8	7.6	21.2	Cytochrome P450, CYP6P4a	13.4	20.9	669.4	115.2	32.5
AFUN015895	3	2.6	2										Cytochrome P450, CYP4H25	86.7	63.7	116.3	304.8	93.6
AFUN005672	2.8	32.7	2.9										cuticular_protein	63.9	35.4	42	157.3	45.5
AFUN009940	2.8	2.8	2.7										cuticular_protein_RR-1_family_(CPR141)	41.3	16.3	21.1	73.7	20.8
AFUN009921	2.5		2.6				2		2.1				cuticular_protein	91.7	8.8	10.5	35.1	22.7
AFUN001383	2.5	2											Cytochrome P450, CYP9J11	465.3	533.3	1230	2113	826.7
AFUN015894	2.4	2	3.3										Cytochrome P450, CYP4H28	6.9	19.5	18	76.2	29.5
AFUN002910	2.4		4.3										lipase member H-like	41.7	52.5	36.8	207.3	36.7
AFUN002772	2.4	2.3								2.1			UDP-glucuronosyltransferase	87.8	40	102.4	156.3	53.4
AFUN008819	2.2	2.1								3.1		2.9	glutathione S-transferase, GSTMS3	70.2	42.1	160.2	149.2	56.3
AFUN015714	2.1						2.9			2.5			Cytochrome P450, CYP6AD1	41	13.8	42.7	46.5	51.1
AFUN008426	2.1	3.6										2.2	glutathione S-transferase, GSTU2	134.3	43.2	71.5	149.2	32.9
AFUN015777	2		2.3										Cytochrome P450, CYP4C26	14.4	34.2	36.9	111.7	57.9
AFUN015961	2	2.9											Cytochrome P450, CYP6Y1	101.6	45.4	60.3	146.6	40.5
AFUN015767	2	2.7								2.1		2.8	glutathione S-transferase, GSTD11	20.2	26.3	68.2	86.5	25.1
AFUN015966		2.3		5.3	12.1	4.5						2.7	Cytochrome P450, CYP325A	14.4	348.3	96.2	105.9	36.6
AFUN008925		4.5			10.2								cuticular_protein_CPLCG_family_(CPLCG5)	145	64.6	133.1	46.5	8.1

AFUN014434	4.4			6.5	2.3			2.8	alcohol dehydrogenase	163.3	121.2	64	132.5	23.8
AFUN004150	4.2			6.4	2.6			2.5	alcohol dehydrogenase	131.2	106.2	51.1	114.3	21.3
AFUN002514			3.8	4.7			2.5	3.3	Carboxylesterase	707.9	3530	2873	1485	964.6
AFUN005677	2.7			4.3				2.9	cuticular_protein_RR-1_family_(CPR81)	846.9	401.3	340.4	405.8	118.9
AFUN008930	3.8			3.8				2.1	cuticular_protein_CPLCG_family_(CPLCG1)	1258	259.1	178	411.4	86.6
AFUN015809			2.6	3.7			3.6	5.2	glutathione S-transferase, GSTe2	471.3	2506	4398	1583	862.8
AFUN015923	3.4			3.5				2.1	aquaporin Z	1860	1784	1345	2735	643.8
AFUN002520				3.3					cuticular_protein	1008	1602	1109	2372	627
AFUN015924	2.7			3.2					aquaporin Z	240.2	311.2	201.2	427.1	123.7
AFUN006309			2.5	2.8					cuticular_protein_CPCFC_family_(CPCFC1)	43.8	26.8	19.4	17.1	12
AFUN010428	2			2.8					Cytochrome P450, CYP4G16	14414	18020	11720	20972	8283
AFUN015810				2.8			2.2	4.1	glutathione S-transferase, GSTE4	757.5	1663	3035	1799	763.3
AFUN002157	2.7			2.7				2.2	glutathione S-transferase, GSTMS2	201.1	161.8	164.3	268.3	77.7
AFUN001429	3.8	3.2		2.6	2.2				alkaline phosphatase	40.9	61.8	34.3	142.6	29.9
AFUN005675				2.6					cuticular_protein_RR-1_family_(CPR79)	200.8	71.6	67.6	83.7	35.3
AFUN008662	2			2.6					Cuticular_protein_unclassified	101.8	56.7	36.2	72.3	28
AFUN009234	2.1			2.6					glutathione S-transferase, GSTU1	425.8	407.7	327.5	540.8	202.4
AFUN010800	3.6	2.2		2.5					cuticular_protein_RR-2_family_(CPR146)	115.4	61.4	49.2	139.3	30.8
AFUN004689			2.6	2.4		2.3	6	5.4	cuticular_protein_RR-2_family_(CPR130)	228.4	57.6	164.1	35.7	31.1
AFUN007144				2.4					alkaline phosphatase	1915	2405	2159	3121	1262
AFUN002517				2.4					Carboxylesterase	1360	1557	1271	1915	
AFUN015723	2.1			2.4					Cytochrome P450, CYP6AH1	537.4	980.2	775.9	1355	510.1
AFUN007143	2.1			2.3					alkaline phosphatase	1080	2434	2021	3442	1320
AFUN000995				2.3					aquaporin	1516	1631	1339	1708	916.6
AFUN005674	2.2			2.3				2.3	cuticular_protein	2214	1188	1468	1844	655.6
AFUN015807				2.3				2.4	glutathione S-transferase, GSTE1	62	196	258.2	249.3	110.4
AFUN007297	2.5			2.2					aldehyde dehydrogenase (NAD+)	1930	2639	2178	4813	1540
AFUN011481				2.1					Cytochrome P450, CYP4G17	2554	2287	2151	3056	1416
AFUN015786	2.5			2				2.7	Cytochrome P450, CYP6AA1	1938	2302	3702	4491	1433

AFUN006221	2.4			2					glutathione peroxidase, GPX4	336.6	157.5	148.3	294.7	98
AFUN008829				2					glutathione S-transferase, GSTS1	4380	3849	3989	5355	2413
AFUN015888			6.9		4.4		6.3		Cytochrome P450, CYP6P5	141.8	808.5	919.3	189.8	661.4
AFUN008925			2.2				3.7	16.9	cuticular_protein_CPLCG_family_(CPLCG5)	145	64.6	133.1	46.5	8.1
AFUN010493			2.1						cuticular_protein_RR-2_family_(CPR113)	926.1	399	367.1	308.4	284.1
AFUN002611					2				alkaline phosphatase	329.8	254	395.5	272.3	434.3
AFUN002021	3	3.2							alpha-amylase	537	339.6	295.5	1224	323.2
AFUN005361	2.6								alpha-amylase	136.1	147.9	173.7	326.4	99.5
AFUN011501	2.9							2.2	Aquaporin	363	136.2	218.9	372.5	101.3
AFUN004002	2.9								Argininosuccinate lyase	3313	4799	4720	11518	3182
AFUN000152	2.4								ATP-binding cassette sub-family A member 3	725.1	512.4	579.8	1222	396.4
AFUN008941	2.4								ATP-binding cassette sub-family C member 1	156.8	254.7	302.8	590.1	193.6
AFUN008942	2.2								ATP-binding cassette sub-family C member 1	686	1047	1152	2190	797.7
AFUN015979	2.2								ATP-binding cassette sub-family G member 2-like	240.9	107.6	122.8	322.5	118.3
AFUN000422	2.8								Carboxylesterase	686.3	1248	820.6	2086	590.1
AFUN001273	2.5								Carboxylesterase	514.2	338.2	433.7	819.9	264.2
AFUN011531	2.3								Carboxylesterase	688.3	371.8	362.7	793.7	276.6
AFUN007079	2.9	2.1							Caspase-9	268.3	482.2	423.9	1142	315.9
AFUN007080	2.9	2.2							Caspase-9	68.6	133.5	132.3	371.9	99.9
AFUN004759	2.2							2.3	cuticular_protein	54.4	30.6	57	69.6	25.4
AFUN006524		5				2.9			cuticular_protein_RR	170.5	16.6	8.3	53.4	25
AFUN005670								2.1	cuticular_protein_RR	5252	1566	2761	3319	1366
AFUN007919	3.3	2.1							cuticular_protein_RR-2_family_(CPR10)	2595	1028	1012	2754	661.2
AFUN015739					2.4				cytochrome P450, CYP307A1	473.1	339.7	470.6	443.5	843.5
AFUN006135	2.2								Cytochrome P450, CYP4C36	222.7	441.5	732.3	842	365.7
AFUN015990	2.3	2.2							Cytochrome P450, CYP4D16	129.4	153.2	120.1	344.6	120.1
AFUN004315	2								Cytochrome P450, CYP4H18	298.7	268	390.2	535.3	207.9
AFUN015785	2.1								Cytochrome P450, CYP6AA2	231.5	350.1	388.8	748.9	278.4
AFUN015790	2.1							2.1	Cytochrome P450, CYP6AG1	1344	1508	2185	2837	1089

AFUN011130	3.4	3.1						Cytochrome P450, CYP6AJ1	73	67.1	47.2	191.2	44.1
AFUN010921	3	2.4						Cytochrome P450, CYP6M1	70.1	52.5	46	144.6	38.4
AFUN010919	2.2							Cytochrome P450, CYP6M1	125.7	131.4	122.7	237.4	87.2
AFUN015795					2		2	Cytochrome P450, CYP6M7	2839	2850	7123	8898	3608
AFUN010918	3.2						2.1	Cytochrome P450, CYP6N1	394.5	622.7	911.3	1824	454.6
AFUN015801	4.7	2.1					2.3	Cytochrome P450, CYP6P2	667.8	561.5	901.2	2418	410.1
AFUN015866	2							Cytochrome P450, CYP9J14	1767	743.4	766.1	1431	555.7
AFUN007549			4.6	2.6	2.6			Cytochrome P450, CYP9K1	1118	1149	3652	3239	6678
AFUN008239	3.1							cytosolic sulfotransferase 3-like isoform X2	190.4	418.9	420.2	1073	270.3
AFUN015768	2.5							glutathione S-transferase, GSTD11	136.4	217.2	281.3	453.6	145.3
AFUN001774	2.1						2	glutathione S-transferase, GSTE7	130.9	166.6	249.3	335.9	127.1
AFUN002155							2.3	glutathione S-transferase, GSTMS1	403	288.6	567.6	547.7	248.6
AFUN004989						2.7		kelch-like ECH-associated 1	662.8	572.1	1666	801.2	968.1
AFUN004354	2.6							UDP-glucuronosyltransferase 1-8	334.4	361	401.8	858.4	264.4
AFUN011266	2.3							UDP-glucuronosyltransferase 3A1	47.2	132.9	141.4	334.8	87.6
AFUN002567							2.1	Xanthine dehydrogenase 1	614.9	340.5	914.7	1151	447.9

MWI=Malawi, GHA= Ghana, CMR= Cameroon and UGA= Uganda, FNG=FANG laboratory susceptible strain
